# Genome-Wide Profiling of the Activity-Dependent Hippocampal Transcriptome

**DOI:** 10.1371/journal.pone.0076903

**Published:** 2013-10-17

**Authors:** Guido Hermey, Claudia Mahlke, Jakob J. Gutzmann, Jörg Schreiber, Nils Blüthgen, Dietmar Kuhl

**Affiliations:** 1 Institute for Molecular and Cellular Cognition, Center for Molecular Neurobiology Hamburg, University Medical Center Hamburg-Eppendorf, Hamburg, Germany; 2 Department of Immunology, Max Planck Institute for Infection Biology, Berlin, Germany; 3 Institute for Theoretical Biology and Institute of Pathology, Charité - Universitätsmedizin Berlin, Berlin, Germany; CNRS UMR7275, France

## Abstract

Activity-dependent gene expression is central for sculpting neuronal connectivity in the brain. Despite the importance for synaptic plasticity, a comprehensive analysis of the temporal changes in the transcriptomic response to neuronal activity is lacking. In a genome wide survey we identified genes that were induced at 1, 4, 8, or 24 hours following neuronal activity in the hippocampus. According to their distinct expression kinetics we assigned these genes to five clusters, each containing approximately 200 genes. Using in situ hybridizations the regulated expression of 24 genes was validated. Apart from known activity-dependent genes our study reveals a large number of unknown induced genes with distinct expression kinetics. Among these we identified several genes with complex temporal expression patterns. Furthermore, our study provides examples for activity-induced exon switching in the coding region of genes and activity-induced alternative splicing of the 3′-UTR. One example is Zwint. In contrast to the constitutively expressed variant, the induced Zwint transcript harbors multiple regulatory elements in the 3′-UTR. Taken together, our study provides a comprehensive analysis of the transcriptomic response to neuronal activity and sheds new light on expression kinetics and alternative splicing events.

## Introduction

Neurons have the capacity to undergo activity-dependent changes in their molecular composition and structure in order to adjust their synaptic strength. Such synaptic plasticity appears to contribute to a variety of physiological and pathological processes in the adult brain including learning and memory, epileptogenesis, response to ischemia, addiction, and neuropsychiatric and neurodegenerative disorders [1], [2], [3]. While short-term activity dependent synaptic changes rely on posttranslational modifications of pre-existing proteins, the long-term maintenance of synaptic adaptations requires gene induction [4]. Signaling from the synapse to the nucleus, which activates gene expression, induces protein synthesis that alters the composition of synaptic protein networks and provides a mechanism for translating synaptic activity into persistent changes of synaptic strength [5], [6].

Much attention has been focused on the identification of genes induced by neuronal activity. In early studies we and others had used unbiased differential screening techniques to identify genes that are transcriptionally induced by seizure activity in the hippocampus [7], [8], [9]. Almost all genes that are known to be induced during long-term potentiation (LTP) were initially identified in such screens and several activity-dependent genes were shown to play important roles in the structural and functional changes underlying long-term plastic events in the nervous system [10]. The development of genome-scale molecular techniques, such as the microarray technology, allows the identification of global changes in expression of a larger set of genes. Microarray screens have been conducted previously to identify activity-regulated genes in the hippocampus, but these experiments were limited because initially developed microarrays represented only an incomplete selection of the transcriptome. Moreover, only a small number of transcripts were confirmed in subsequent validation experiments, and most studies did not attempt to monitor a time course of transcriptional induction following synaptic stimulation [11], [12], [13], [14]. In addition, information on activity-dependent alternative splicing is scarce. Thus, it is likely that the majority of activity-regulated genes and their post-transcriptional regulation remain to be discovered.

Here we revisit the unbiased identification of activity-dependent genes using genome-wide microarray profiling. We report a systematic survey of alterations in gene expression in the hippocampus at four different time points following patterned neuronal activity. DNA microarrays representing the entire transcribed mouse genome were used to examine large-scale changes in gene expression. We identified 5 groups of highly induced genes with different kinetics of induction and each group comprising more than 200 genes. Moreover, our study reveals that exon switching and alternative 3′-UTR usage are frequently occurring mechanism in response to neuronal activity.

## Materials and Methods

### Tissue Preparation

3 month old male mice were injected with kainic acid or isotonic saline solution according to institutional guidelines. Kainic acid (16 mg/kg, Ascent scientific) or similar amounts of isotonic saline were administered by i.p. injection. Animals were sacrificed by cervical luxation 1, 2, 4, 8, or 24 h after onset of the first seizure (each time point, n = 3), control animals were sacrificed 1 h after saline injection (0 h, n = 6).

### Microarray Hybridization

For RNA isolation hippocampi were dissected from fresh brains, flash frozen and stored at −80°C. Total RNA was isolated using TRItidy-reagent (Applichem), followed by an additional purification step using RNEasy columns (Qiagen), quantified by UV-spectroscopy and its quality verified using a LabChip BioAnalyzer (AGILENT Technologies). The amplification and labeling of RNA samples were conducted according to the manufacturer’s instructions (Affymetrix). One µg from each sample was transcribed to cDNA using an oligo(dT)24 primer containing a T7 RNA polymerase promoter. After RNAse H-mediated second strand cDNA synthesis, the product was purified and served as a template in the subsequent *in vitro* transcription reaction. Biotin-labeled cRNA was prepared from double-stranded cDNA by *in vitro* transcription using the GeneChip RNA transcript labeling kit (Affymetrix). After cleanup, biotin-labeled cRNA was fragmented by alkaline treatment [40 mmol/l Tris-acetate (pH 8.2), 100 mmol/l potassium acetate, and 50 mmol/l magnesium acetate] at 94°C for 35 minutes. Fifteen µg of each cRNA sample was hybridized for 16 h at 45°C to an Affymetrix mouse genome 430 2.0 GeneChip containing 45.000 probe sets and covering the complete transcribed mouse genome. Chips were washed and stained with streptavidin-phycoerythrin using a fluidics station according to the protocols recommended by the manufacturer. Finally, arrays were scanned at 1.56-µm resolution using the Affymetrix GeneChip System confocal scanner 3000.

### Data Analysis

Data from GeneChip microarrays has been deposited in the NCBI Gene Expression Omnibus (GEO) and is accessible through the GEO Series accession number GSE49030. In addition, we have generated an online database that allows one to search for genes of interest. All microarray data analysis was performed using bioconductor available in the statistical package R (http://www.bioconductor.org/), using the packages affy, annaffy and biomaRt. Expression values were calculated using the Robust Multichip Average (RMA) method. The variance of biological replicas was estimated from the 6 arrays hybridized with samples from control mice. Using loess with standard parameters, an error-model was established for the standard deviation as function of mean expression. This error-model was used to assign a Z-value for each gene at each time-point by dividing the difference in expression through the standard deviation obtained from the error model. Genes that were induced with a Z-value >5 at any time point and had a MAS5 (Affymetrix) “present” call in at least 3 arrays were defined as significantly regulated and subjected to k-means clustering with 5 clusters. Using 25 random permutations of the untreated samples, we estimated the false discovery rate of this procedure to be below 0.05. Gene Ontology analysis was performed using Gossip (gossip.gene-groups.org), K-boxes were identified using the Transterm search facility [15], overrepresented transcription factor binding sites were searched using Transfind with standard parameters [16]. Comparison with other data sets, and conversion to homologous genes was done using Ensembl via the biomaRt package in R, on the basis of Entrez gene ids.

### *In situ* Hybridization

Total brains were flash frozen using liquid nitrogen and stored at −80°C until cryosectioning. Antisense RNA probes labeled with [alpha-^35^S]-UTP were generated according to the manufacturer’s instructions (Promega). Twenty µm cryosections of brains were fixed in 4% paraformaldehyde-PBS, acetylated, dehydrated and hybridized at 55°C for 18 h. Ribonuclease A treatment was performed for 30 min at 37°C. Following a high stringency wash in 0.1×saline sodium citrate buffer at 55°C, slides were exposed to X-ray films (Kodak Biomax MR; Amersham Bioscience) for 72 h. Specificity of signals was verified by comparing antisense to sense controls. DNA templates were generated by PCR or restriction digest from full-length cDNA clones and cloned into pBSK (Stratagene). For a complete list of templates see Table 1. Autoradiograms were imaged with a Leica stereo microscope and densitometric analysis was performed using ImageJ software (http://rsb.info.nih.gov./ij). A region of interest (ROI) encompassing the entire hippocampus was analyzed in two independent samples for each time point of a seizure series (0 h, 1 h, 2 h, 4 h, 8 h and 24 h). Average gray values were background corrected and normalized to the 0 h time point of each series.

**Table 1:** Genes used for *in situ* hybridizations, respective nucleotides of templates and accession numbers.

Gene	Nucleotides	GeneBank™ Accession no.
Arc	589–1198	NM_018790
Arf2	757–1991	NM_007477
Arfl4	466–1306	NM_025404
Arl5b	760–2400	NM_029466
ErcI	3614–4281	NM_053204
ErcII	4395–5144	NM_177814
Errfi1	255–2317	NM_133753
Gem	114–1834	NM_010276
GPR19	741–1376	NM_008157
GPR22	1857–2963	AK048077
GPR84	1–985	BC023249
GPR115	1638–3050	BC089564
Homer1a	1191–2136	NM_011982
NPY	1–491	BC043012
Rasl10a	1–1009	BC107200
RhoJ	1–448	BC060193
Rnd3	314–1676	NM_028810
Rrad	274–1191	NM_019662
Rras2	786–1796	NM_025846
Siah1a	430–1129	BC046317
Siah1b	630–1787	BC052887
Siah2	1–1194	BC058400
SorCS3	1825–2504	AF276314
Tubb6	1336–1736	BC008225
Zwint (var.1)	815–1516	BC034870
Zwint (var. 2)	824–1567	NM_025635

## Results

### Identification of Activity-regulated Genes

**Figure 1:**
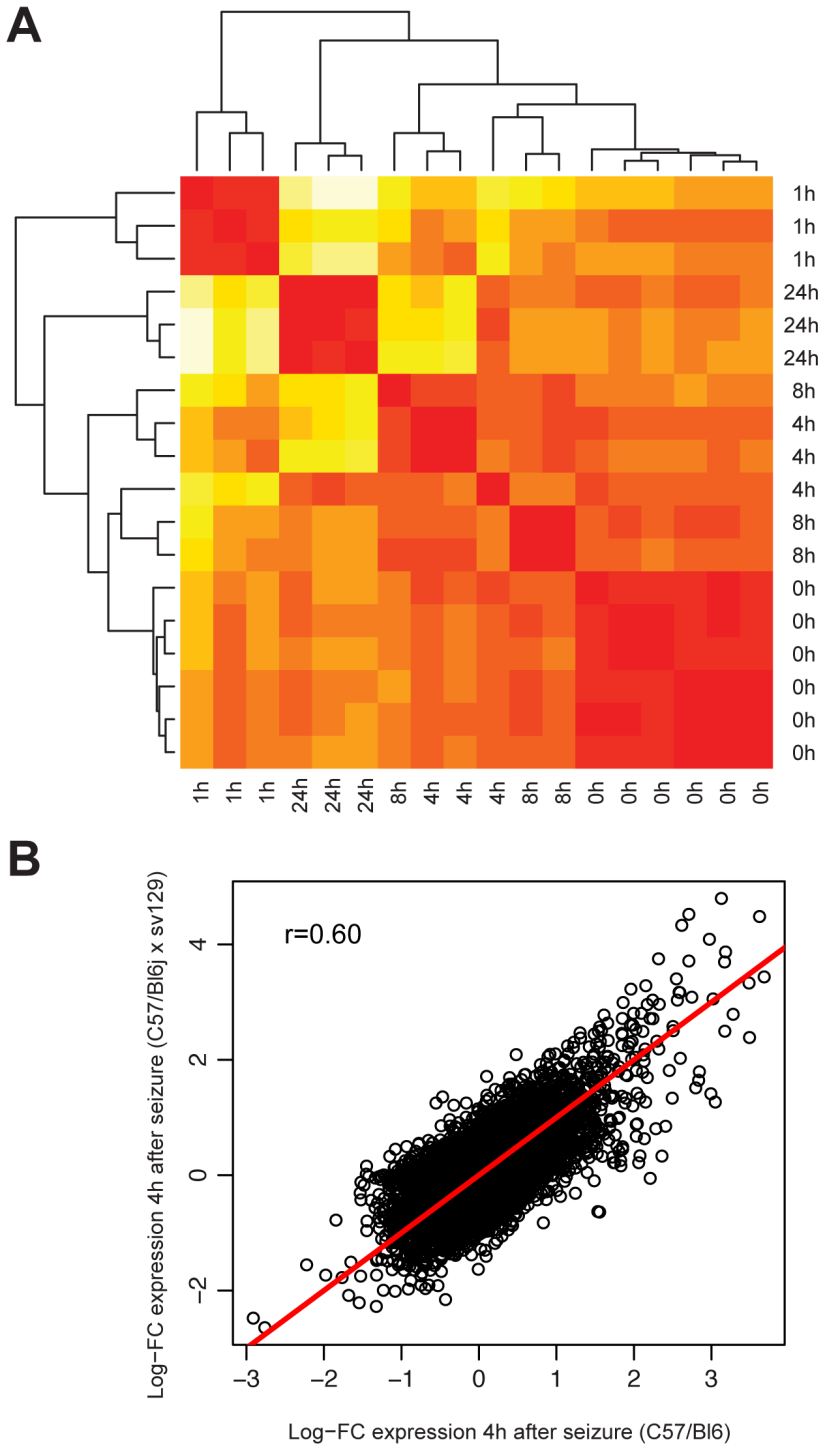
Correlation of activity induced genes. **A,** Two-dimensional correlation plot of activity induced gene expression levels. Six independent experiments were conducted for the 0 h control time point and three independent experiments for the time points 1, 4, 8, and 24 h. Hippocampal RNA of one mouse was hybridized to one DNA microarray. The plot indicates the correlation of expression profiles of all induced genes compared in control animals and in animals at indicated time points after the onset of kainic acid induced seizures. Correlation coefficients range from 0.97 (bright yellow color code) to 0.99 (red color code). Arrays were clustered using average linkage cluster analysis with a correlation metric distance. **B**, Correlation of genes induced by seizure in mice of different genetic backgrounds. Hippocampal RNA from control mice and mice sacrificed 4 h after the onset of seizure was analyzed. RNA from one animal (n = 3) was hybridized to one microarray and genes induced 4 h after onset of seizures in C57Bl/6J and hybrid 129/Sv×C57Bl/6J mice were compared. Logarithmic fold changes (Log-FC) are shown. Correlation coefficient r = 0.6 is indicated.

We triggered seizures to induce strong synchronous neuronal activity in the brain and obtained hippocampal tissue for microarray analysis from animals sacrificed 1, 4, 8, or 24 h after the onset of seizures. RNA extracted from one hippocampus was hybridized to one microarray, allowing us to reliably assess biological variability. We compared gene expression levels of 6 control mice and found them to be almost identical (r = 0.99). Comparing the expression profiles of all activity-induced genes in all animals revealed a high correlation of these profiles with very low inter animal variability (Fig. 1A).

Differences in the vulnerability to seizure-induced excitotoxicity between different mice strains were previously reported [17]. Here we used C57Bl/6J mice, which do not display hippocampal neurodegeneration frequently observed in other strains of mice following kainic acid induced seizures. First, we investigated if genetic variations may give rise to differences in the activity-regulated transcriptome in mice of different genetic background. We compared the induction of genes 4 h after the onset of seizures between C57Bl/6J and hybrids of 129/Sv×C57Bl/6J and found a high correlation of induced genes in both genetic backgrounds, indicating that the changes in activity-regulated gene expression are generally not strain specific (Fig. 1B).

**Figure 2:**
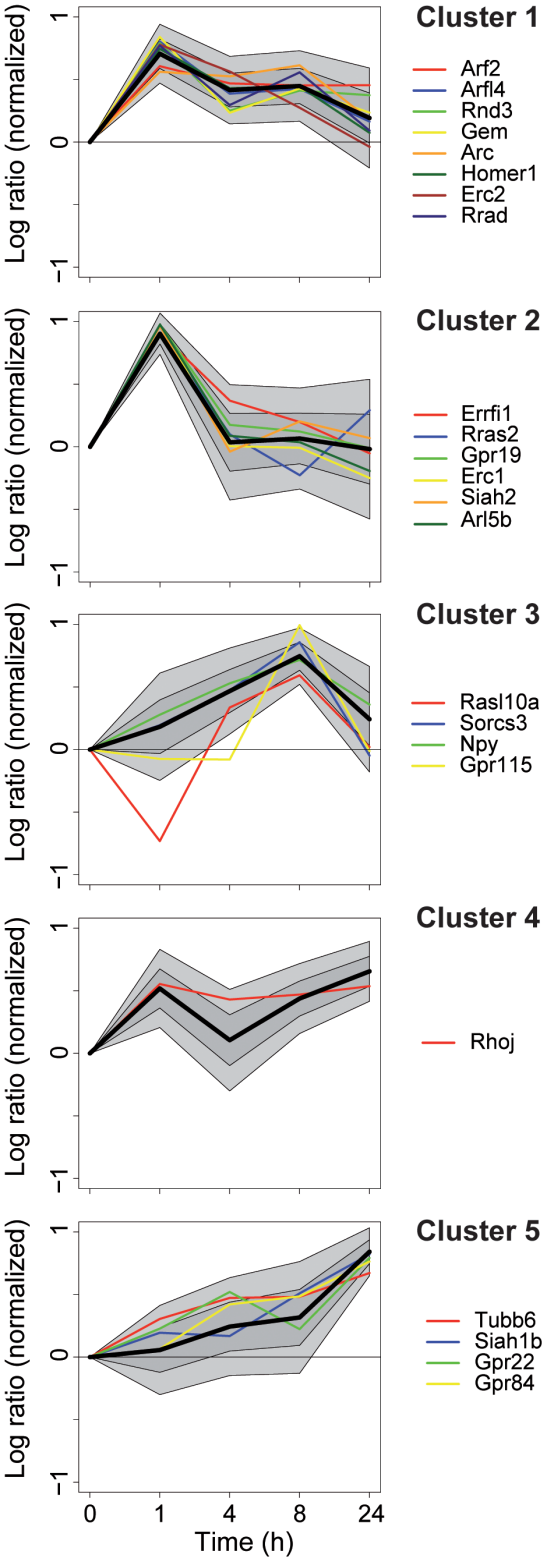
Expression profiles of genes assigned to one cluster. Log ratios were normalized by the Eukledian distance to 0. Black lines show the average normalized log ration. Dark and light grey areas indicate one and two standard deviations, respectively. Genes used for validation experiments are highlighted in color. The 254 transcripts of cluster 1 showed strong induction 1

**Figure 3:**
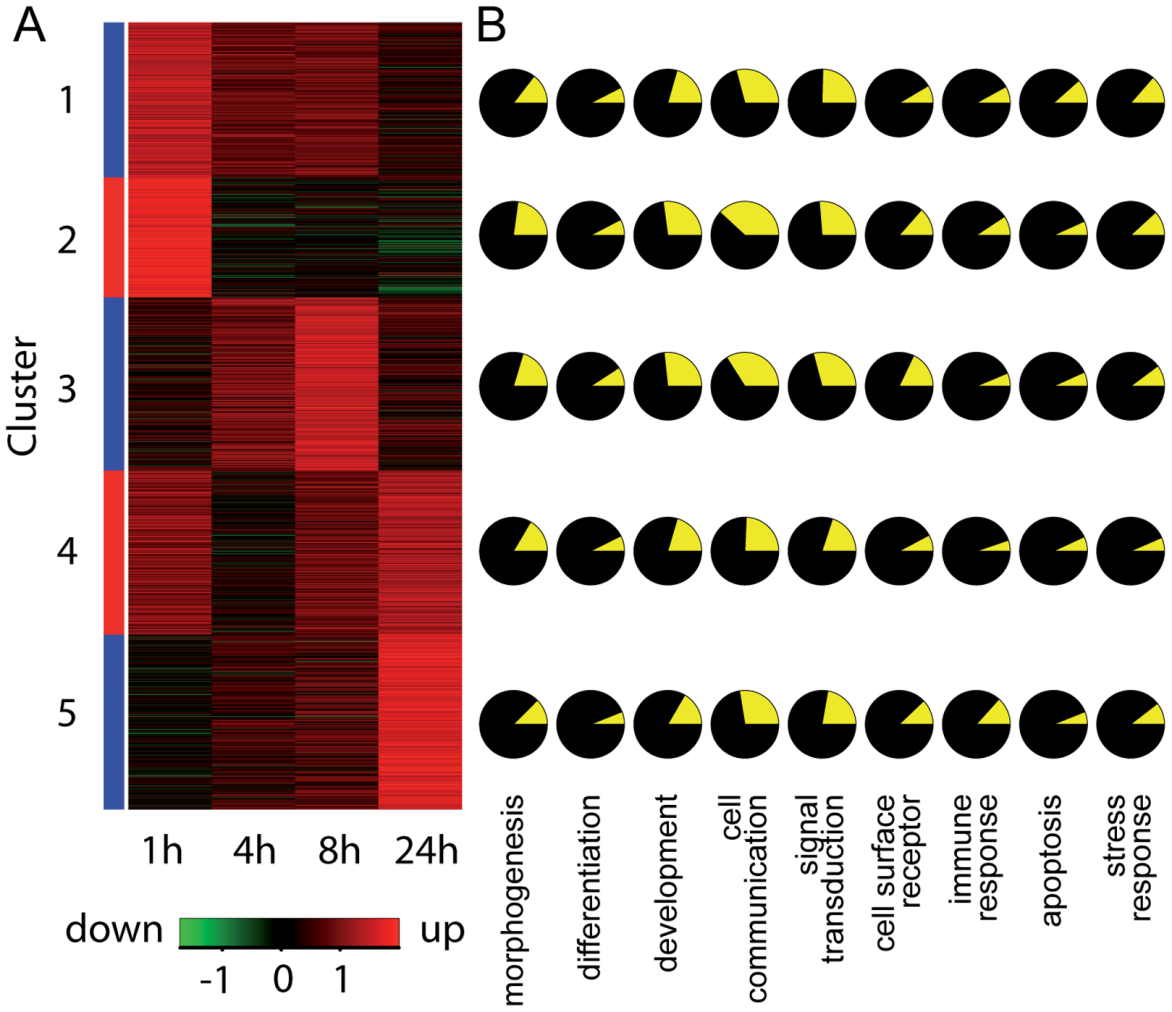
Gene expression profiles induced by neuronal activity and a functional categorization of identified genes. **A,** Heat map providing an overview of different time courses of expression induced by neuronal activity. Transcripts were clustered into five groups according to their time course of expression. Red indicates upregulation, green indicates downregulation. **B,** Categorization of biological functions of the genes belonging to the identified clusters according to the Gene Ontology database. The fraction of genes of a classified function is shown in yellow.

We next set out to identify genes that are robustly induced by neuronal activity. We used the following criteria to define a gene as induced (i) the corresponding probe set had to have a MAS5 (Microarray Suite 5 method) “present” call in half or more samples, (ii) a change in expression had to be more than fivefold over standard deviation, which was estimated by averaging the standard deviation of genes with similar expression level. Using these settings, we estimated that less than 5% of the identified genes are false positives. Following our definition we identified 1186 genes induced by neuronal activity in the hippocampus of C57Bl/6J mice. Apart from known activity-dependent genes our study reveals a large number of genes that have not been previously described as induced by neuronal activity in the hippocampus. We used an unsupervised clustering algorithm and chose five clusters, because this was in agreement with a small number of groups and group homogeneity. According to the time course of expression we defined clusters with a specific transcriptional profile (Fig. 2), each comprising about 200 transcripts (Fig. 3A). We used the Gene Ontology (GO) database to relate genes to biological processes in which the respective gene products participate (Fig. 3B). Of the identified 1186 genes 982 encode proteins whose functions have been listed in GO categories. The complete list of genes is given in the table S1.

### Validation of Selected Sets of Genes

From the microarray data set we tested 20 genes that had not been previously identified as activity-regulated in independent *in situ* hybridization experiments. In addition we included as positive controls four previously described activity-regulated genes. Transcriptional changes were analyzed at 1, 2, 4, 8, and 24 h after the onset of seizures. Each time point was tested on sections of three animals. The specific probes for each gene were selected independently of the probe sets of the microarrays. Among the tested genes were the orphan hepta-helical receptors GPR19, GPR22, GPR84, and GPR115 and the mitogen-induced gene Errfi1/Mig6 all of these are potentially involved in signal transduction. Siah1 and Siah2 are E3 ubiquitin ligases related to proteasomal function. Arf2, Arfl4, Arl5b, Gem, Rnd3, RhoJ, Rrad, Rras2, Rasl10a, Tubb6, Zwint have a predicted or established function in intracellular trafficking and cytoskeleton dynamics. Erc1 and Erc2 (also named Cast2 and Cast1) have been previously described to play important roles in the organization of the presynaptic active zone.

**Figure 4:**
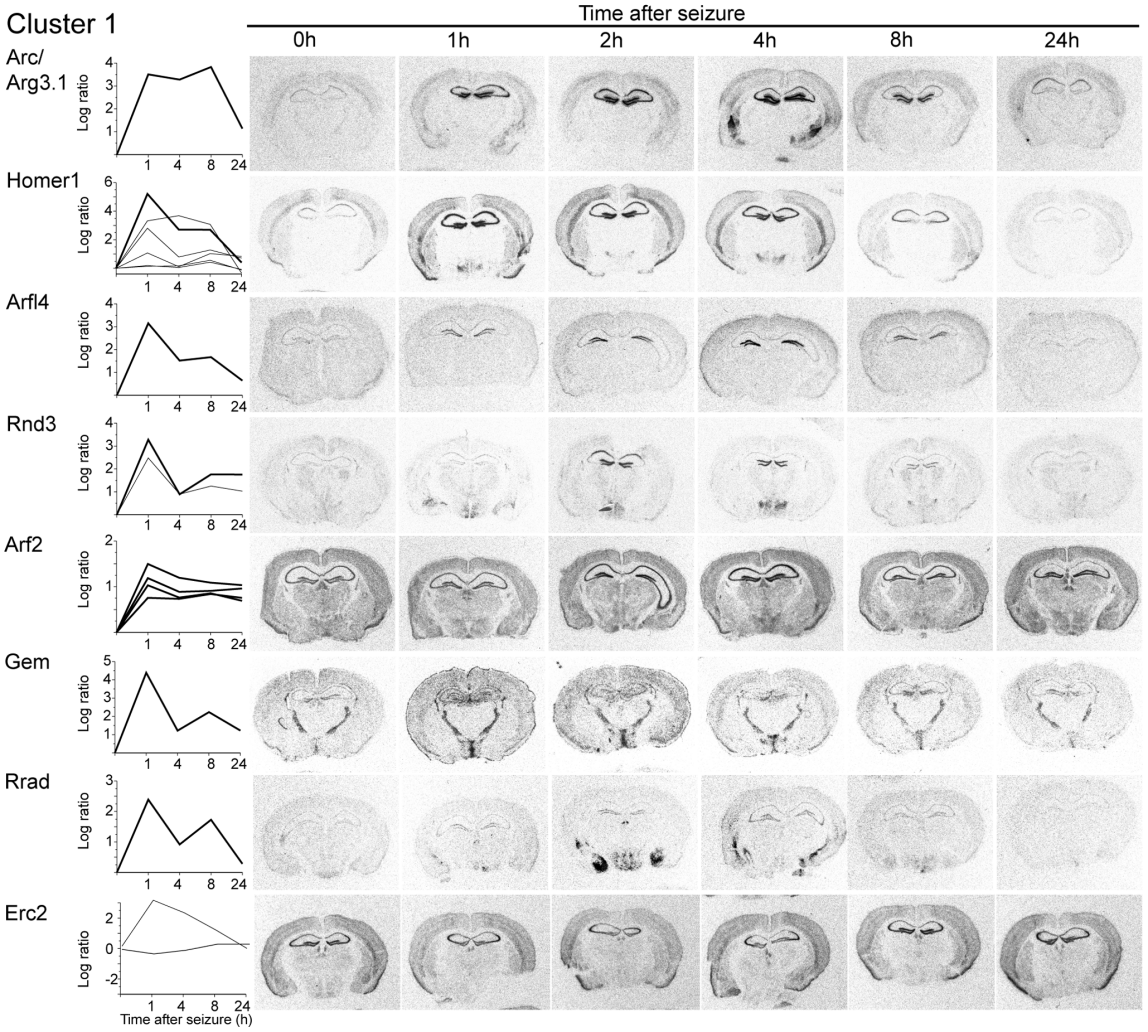
Examples of the temporal and spatial expression of activity-regulated genes of cluster 1. (Left) Plots of the time course and the ratio of induction based on the microarray analyses. Shown are expression profiles of genes represented by one or more microarray probe sets. A bold line indicates the microarray probe set that corresponds to the probe used in the respective *in situ* hybridization analysis. (Right) Autoradiograms of coronal sections of mice of different time points following seizure. Radioactive *in situ* hybridizations of sections were conducted in parallel on one glass slide using gene specific antisense RNA probes. Note: The fragment used for *in situ* hybridization to show Erc2 induction does not correspond to any of the probe sets, therefore none was marked with a bold line (compare Figure 9).

As positive controls we used Arc/Arg3.1, Homer1, Neuropeptide Y (NPY) and SorCS3. Arc/Arg3.1 is essential for consolidation of plasticity and memories [18], [19] and has been implicated in intracellular postsynaptic trafficking and F-actin expansion [20], [21], [22]. Homer1 is a scaffold protein of postsynaptic densities of excitatory synapses [23]. Arc/Arg3.1 and Homer1 expression is prototypic for genes assigned to cluster 1, as their expression is rapidly induced within the first hour following the onset of seizure and expression levels remain still elevated after 8 h [23], [24], [25]. This expression kinetic is seen in our microarray as well as in the *in situ* hybridizations analyses (Fig. 4). Differences in the expression kinetics are seen at the 8 h time point for Arc/Arg3.1 (Fig. 4) and are most likely due to interanimal variability (Fig. 1A). All six tested, previously undescribed activity-regulated genes were confirmed in the *in situ* hybridization analysis to be activity-regulated and to belong to cluster 1. The time courses of induction for Arfl4 and Rnd3 observed in the microarray analysis differed only slightly from that seen in the *in situ* hybridization analysis (Fig. 4 and S1A).

**Figure 5:**
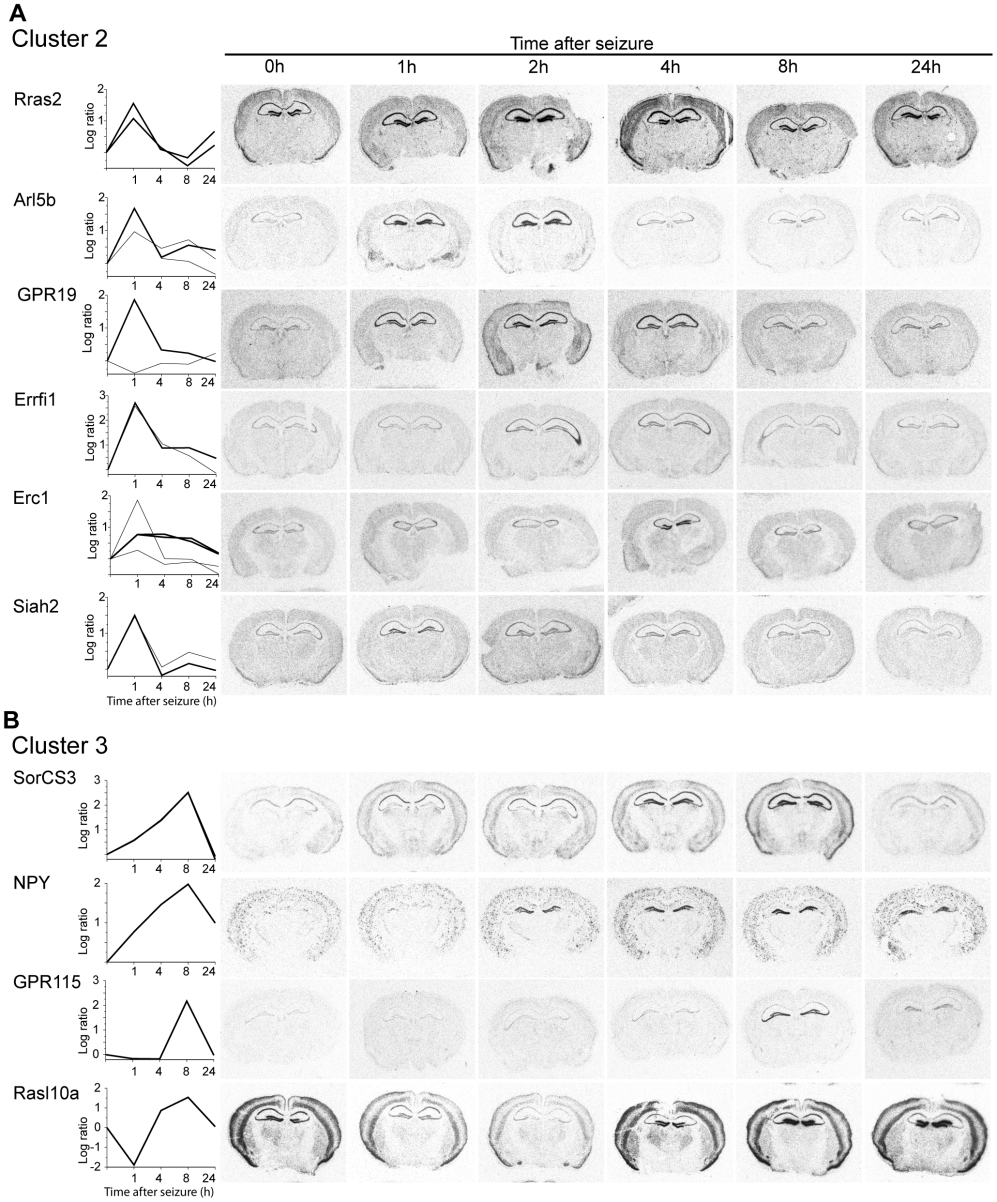
Examples of the temporal and spatial expression of activity-regulated genes of cluster 2 (A) and cluster 3 (B). (Left) Plots of the time course and the ratio of induction based on the microarray analyses. Shown are expression profiles of genes represented by one or more microarray probe sets. A bold line indicates the microarray probe set that corresponds to the probe used in the corresponding *in situ* hybridization analysis. (Right) Autoradiograms of coronal sections of mouse brains at different time points following seizure. Radioactive *in situ* hybridizations of sections were conducted in parallel on one glass slide using gene specific antisense RNA probes. Note: Erc1 was grouped to cluster 1 because of the maximal induction of one probe set after 1 h. The fragment used for *in situ* hybridization does not correspond to this probe set. It matches to the probe sets presented as bold lines.

Genes grouped to cluster 2 were similarly induced 1 h after onset of seizure but their expression levels declined faster to baseline compared to those of cluster 1 (Fig. 5A and S1B).

Expression profiles in cluster 3 exhibit maximal induction 8 h after seizure. Two previously described activity-regulated genes that match the criteria for cluster 3 were analyzed. SorCS3 is a putative intracellular sorting receptor [26]. The induction of SorCS3 transcription observed in the microarray analysis was confirmed in the *in situ* hybridization experiments (Fig. 5B and S2A) and follows the time course we previously reported [27]. NPY expression has been implicated in neuronal functions, anxiety, memory consolidation and cognition [28]. Under conditions of elevated neuronal activity NPY transcript levels are increased and the peptide is released from neurons [28]. Our *in situ* hybridization analysis confirms the time course of expression observed in the microarray studies, with highest NPY expression levels 8 h after the onset of seizure (Fig. 5B and S2A). Among the newly identified activity-dependent genes within cluster 3 is the orphan G-protein coupled receptor GPR115. The *in situ* hybridizations corroborate the array data. GPR115 transcripts are almost undetectable under control conditions and during the first 4 h after the onset of seizure, but are strongly induced at the 8 h time point. Induction of transcript levels is confined to the hippocampal area CA3 and the dentate gyrus (Fig. 5B and S2A). Another example of a newly identified activity-dependent gene is Rasl10a, a RAS-like family member. Rasl10a shows a complex time course of expression. Microarrays and *in situ* hybridization analysis demonstrate that in the hippocampus and cerebral cortex expression levels are significantly down-regulated 1 and 2 h after seizure. Rasl10a expression levels return to base line after 4 h and are subsequently up-regulated with a maximal expression level in the dentate gyrus 8 h after seizure (Fig. 5B and S2A).

**Figure 6:**
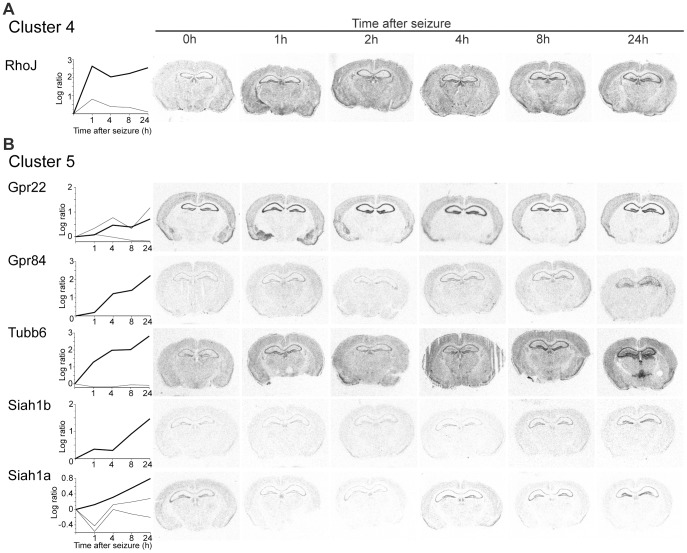
Examples of the temporal and spatial expression of activity-regulated genes of cluster 4 (A) and cluster 5 (B). (Left) Plots of the time course and the ratio of induction based on the microarray analyses. Shown are expression profiles of genes represented by one or more microarray probe sets. A bold line indicates the microarray probe set that corresponds to the probe used in the corresponding *in situ* hybridization analysis. (Right) Autoradiograms of coronal sections of mouse brains at different time points following seizure. Radioactive *in situ* hybridizations of sections were conducted in parallel on one glass slide using gene specific antisense RNA probes.

Genes grouped to cluster 4 follow a biphasic induction profile with maximal expression levels at 1 and 24 h after seizure onset and a reduction of expression levels after 4 h (Fig. 3A). Changes in transcript levels in this cluster are not as pronounced as in the other clusters and induction observed in the *in situ* hybridization analysis was modest (Fig. 6A and S2B).

Expression profiles in cluster 5 are characterized by continuous and moderate induction with a maximal expression level 24 h after seizure. The *in situ* hybridization analyses are in accordance with the expression profiles observed in the microarray survey (Fig. 6B and S2C). We found the E3 ubiquitin ligase Siah1b was significantly induced. The transcript is 97% homologous to Siah1a, which did not match our induction criteria in the microarray analysis and was therefore not assigned to one of the defined clusters. However, our analysis suggests that Siah1a expression is also moderately induced (Fig. 6B). In conclusion, *in situ* hybridization analyses of 26 randomly selected genes confirm the induction patterns observed in our microarray analysis and indicate a low false-discovery rate. Highest changes in expression levels were observed in cluster 1, 2, and 3, whereas gene induction was more moderate in cluster 4 and 5. Induction of gene expression within one cluster follows comparable distinct kinetics, but our *in situ* hybridization analysis reveals differences in the precise anatomical location of induction within one cluster.

### Activity Induced Switch of Exons

**Figure 7:**
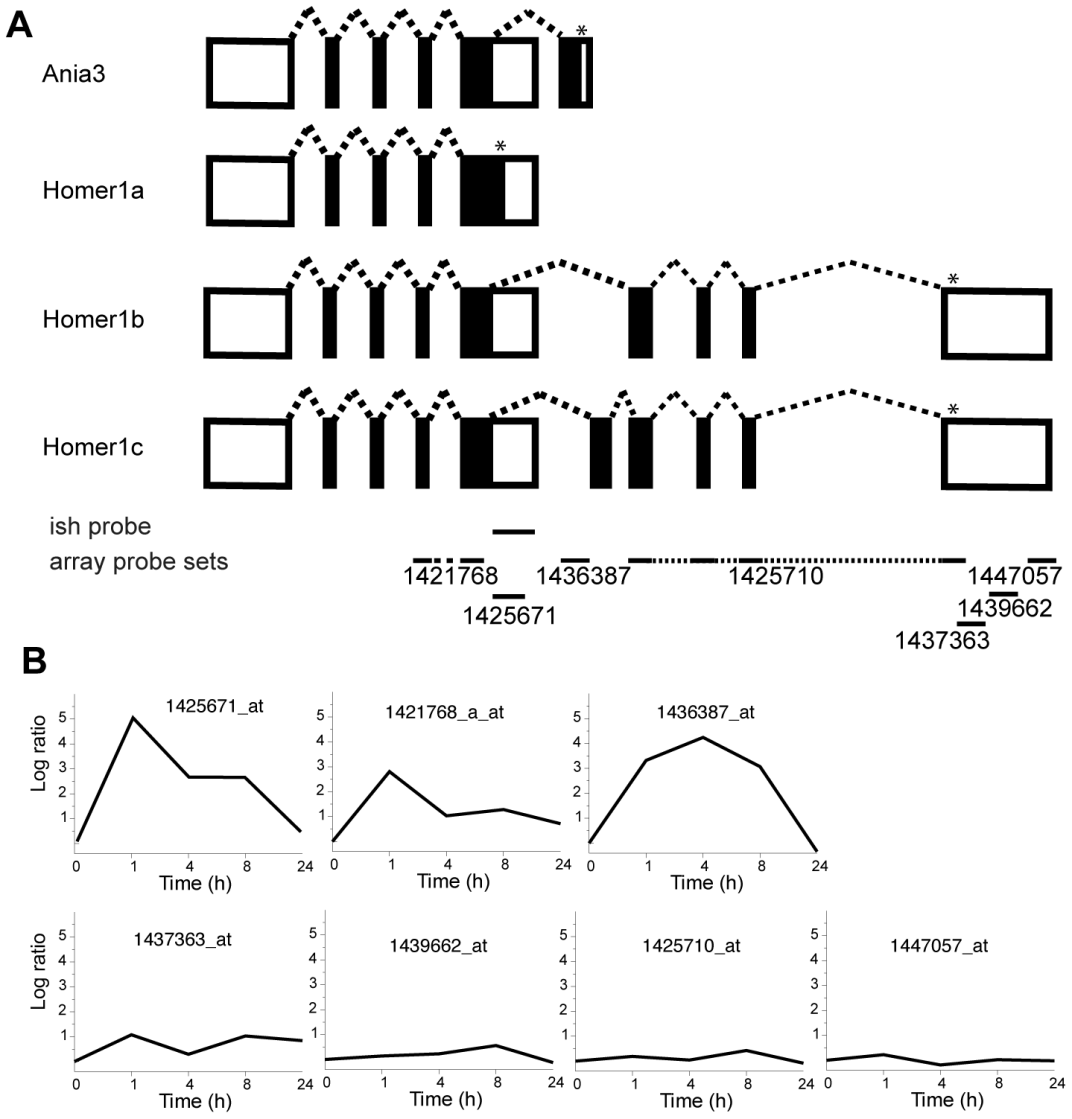
Activity-dependent induction kinetics of Homer1 splice variants. **A,** Genomic organization of the Homer gene. Four splice variants of Homer1 are expressed in the mouse. Exons are depicted as boxes, coding sequence are shown in black and untranslated regions in white. The stars indicate alternative stop codons. Horizontal lines indicate the position of the RNA probe used for *in situ* hybridization (ish) and the microarray probe sets. The numbers of microarray probe sets are indicated. **B,** Expression profiles of Homer using the indicated probe sets in the microarray analysis are shown. The corresponding *in situ* hybridizations are shown in Figure 4.

**Figure 8:**
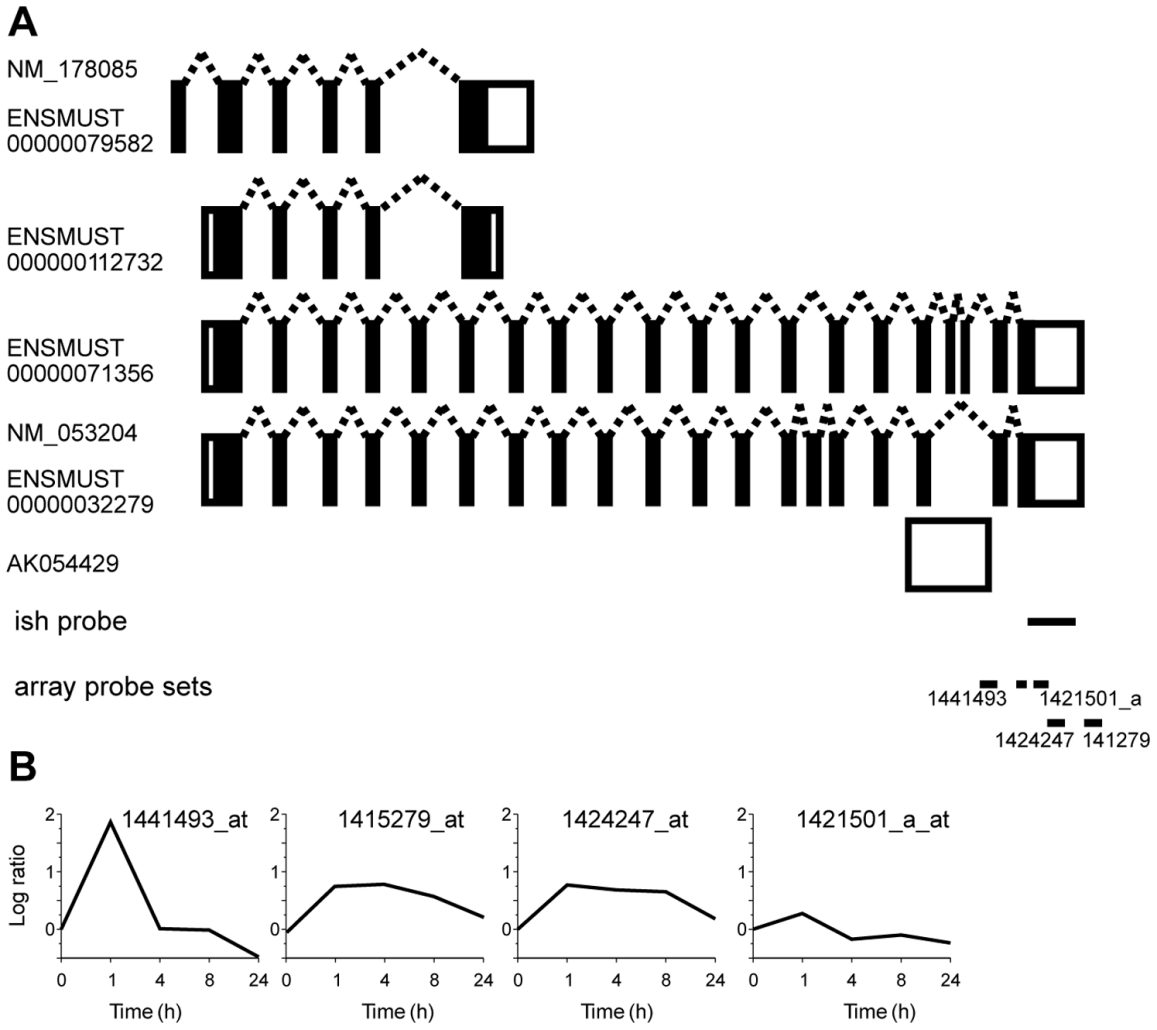
Distinct activity-dependent induction of Erc1 splice variants. **A,** Alternative exon usage of Erc1 transcripts. Four splice variants of Erc1 are expressed in the mouse. Respective Genbank and Ensembl accession numbers are indicated. Exons are depicted as boxes, coding sequences are shown in black and untranslated regions in white. Exon sizes and distances are not to scale. The annotated cDNA AK054429 corresponds to exon 18, 19, and 20 and the surrounding genomic sequence. Therefore it may represent an unspliced transcript or a 3′-UTR of an unidentified splice variant. Horizontal lines indicate the position of the probe used for *in situ* hybridization (ish) and the microarray probe sets. The numbers of microarray probe sets are given. Note that probe set 1441493_at corresponds only to sequence in AK054429. **B,** Expression profiles of Erc1 using the indicated probe sets in the microarray analysis are shown. The corresponding *in situ* hybridizations are shown in Figure 5A.

**Figure 9:**
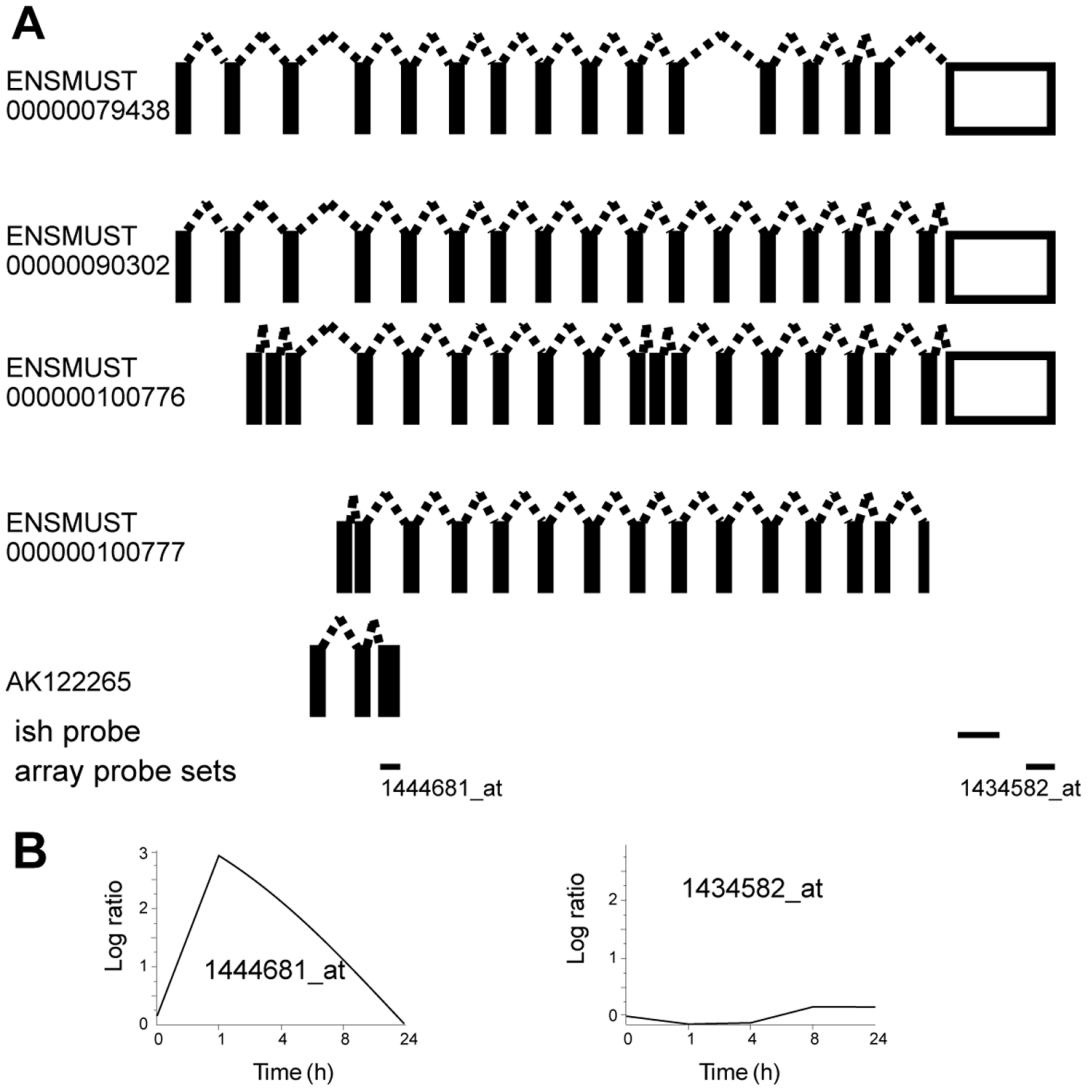
Distinct activity-dependent induction of Erc2 splice variants. **A,** Alternative exon usage of Erc2 transcripts. Four splice variants of Erc2 have been reported in the mouse. Respective Ensembl accession numbers are indicated. Exons are depicted as boxes, coding sequences are shown in black and untranslated regions in white. Exon sizes and distances are not to scale. The annotated cDNA AK122265 corresponds to exon 5, 7, and a novel exon. Therefore it may represent a novel splice variant. Horizontal lines indicate the position of the probe used for *in situ* hybridization (ish) and the array probe sets. The numbers of array probe sets are given. Note that probe set 144681_at corresponds only to sequence in AK122265. **B,** Expression profiles of Erc2 using the indicated probe sets in the microarray analysis are shown. The corresponding *in situ* hybridizations are shown in Figure 4.

Alternative splicing is an important mechanism to regulate and diversify gene functions. One of the few well-studied examples of activity-dependent alternative splicing is Homer1. Neuronal activity induces an exon switch in the Homer1 transcript resulting in an activity-induced alteration of the coding region [23], [29], [30]. Four splice variants have been described of which two, Homer1a and Ania3, were identified as activity-regulated transcripts. Microarrays used in this study contained seven probe sets corresponding to Homer1 and we observe an activity-dependent splicing pattern identical to what was reported previously (Fig. 4 and 7). Probe sets specific for the constitutive splice variants Homer1b and –1c exhibited no induction and a probe set common to all splice variants showed moderate induction. In contrast, we find that expression levels of Homer1a and Ania3 were markedly induced but differed in their time course of induction (Fig. 4 and 7). These data are in complete agreement with previously published data using RNase protection and Northern blot assays to assess transcript levels following electroconvulsive seizures [29]. We were therefore confident that our microarray study could be used to identify unknown activity-dependent splicing events. We examined genes that were represented by more than one probe set and exhibited activity-regulated transcriptional profiles. One example is Erc1 (Fig. 5A). Five alternative Erc1 transcripts can be found in the Ensemble database. Two short and two long variants and a partial transcript (Fig. 8). In our microarray study Erc1 was represented by four probe sets. Of these one probe set exhibited maximal induction 1 h after onset of seizure and showed subsequently a fast decline to baseline levels. Two other probe sets revealed a slower time course of induction, which is in good agreement with our *in situ* hybridization analysis (Fig. 5A and 8). The probe sets showing slow induction correspond to the last exon of the two long splice variants. The probe set revealing a rapid induction corresponds to the partial transcript that most likely represents an uncharacterized activity-regulated short splice variant of Erc1. Another activity-regulated gene identified in our study is Erc2 (Fig. 4). The genomic structure of Erc2 is complex and 5 splice variants have been described (Fig. 9). One probe set for Erc2 showed maximal induction 1 h after seizure and matches an exon present only in one transcript (Fig. 9). A second probe set corresponding to the last exon indicated small changes in expression 8 h after seizure onset. In situ hybridizations in which we used a nucleotide probe from this exon confirmed the moderate induction in the dentate gyrus at this time point (Fig. 4). These examples illustrate activity-dependent splicing events, which result in alterations of the amino acid composition of the translated protein. These alternative isoforms of the constitutively expressed proteins may serve alternative functions and might antagonize or repress the function of the constitutive isoform.

### Activity-induced Selection of 3′-UTRs

**Figure 10:**
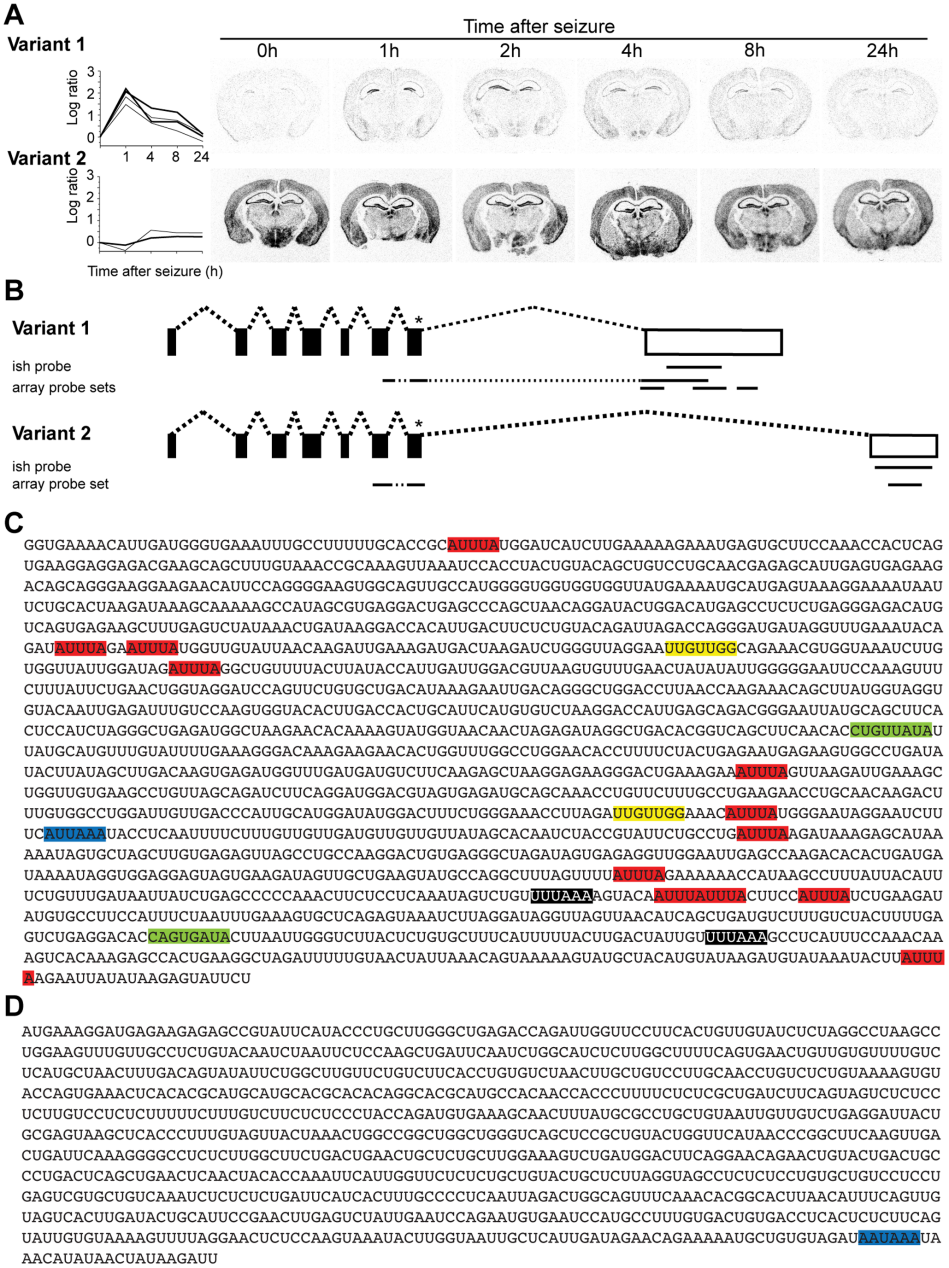
Specific induction of a splice variant of Zwint. **A,** Two alternative splice variants of Zwint are expressed in the mouse. Expression profiles of both variants observed by microarray analysis (left) and by *in situ* hybridization (right) are shown. The plots of the microarray probe sets corresponding to the fragment used for *in situ* hybridization are presented as bold lines. Autoradiograms of coronal sections of mice of different time points following seizure. Radioactive *in situ* hybridizations of sections were conducted in parallel on one glass slide using Zwint splice variant specific antisense RNA probes. **B,** Genomic organization of the Zwint gene. Shared exons are depicted as black boxes, alternatively used 3′-UTRs as white boxes. Stars indicate stop codons, black lines the RNA probes used for *in situ* hybridization (ish) and microarray probe sets. Exon sizes and distances are not to scale. **C, D,** Nucleotide sequence of the last exon of Zwint splice variant 1 **(C)** and splice variant 2 **(D)**. Putative regulatory elements are indicated. AU-rich elements (AUUUA) in red, U-rich motifs (UUUAAA) in black, K-boxes in green, UUGUUGG(G) motifs in yellow, polyadenylation signals in blue.

Activity-dependent splicing affecting the 3′-UTR will result in mRNAs encoding identical proteins, but transcripts may differ in cis-regulatory elements. Here, we identified and analyzed alternative exon usage in Zwint/SIP30 (ZW10 interactor/SNAP25 interacting protein) as an example of activity-dependent splicing affecting the 3′-UTR. Out of 6 probe sets corresponding to Zwint, 4 showed an induction, whereas 2 did not (Fig. 10 A). The murine gene harbors 9 exons and is expressed in two splice variants (Fig. 10 B). The first 7 exons comprise the complete coding sequence and are present in both splice variants. The two terminal exons represent mutually exclusive 3′-UTRs. All induced probe sets correspond to the 3′-UTR of splice variant 1, while the unchanged probe sets correspond to the 3′-UTR of splice variant 2. *In situ* hybridization analysis using specific probes corresponding to the respective 3′-UTRs corroborated these observations (Fig. 10 A). These data demonstrate that variant 2 is constitutively expressed throughout the brain and seizures have only a minor effect on its expression level. In contrast, expression of splice variant 1 is almost undetectable under control conditions, but is markedly induced in the dentate gyrus 1 and 2 h after seizure. We found no apparent regulatory elements in the 3′-UTR of variant 2 controlling the stability of this transcript, whereas the 3′-UTR of the activity-regulated splice variant 1 contains 12 AU-rich elements (AREs), two U-rich motifs (URMs) and two K-boxes, implicated in the regulation of mRNA stability [31], [32], [33]. In addition, we indentified two UUGUUGG(G) motifs which have been suggested to direct activity-dependent polyadenylation [34] (Fig. 10 C, D).

### Transcriptional Control of Activity-regulated Genes

We analyzed whether the promoter regions of the co-regulated genes of one cluster show a significant enrichment of putative high-affinity binding sites for transcription factors [16]. We found several motifs that were over-represented in defined clusters (Table 2). Cluster 1 and 2 show an enrichment of motifs recognized by CREB and other members of the ATF transcription factor family that have roles in transcriptional control of activity-regulated genes [5], [35]. In addition, TBX5, EGR-1 and SP1 binding motifs were also enriched in these clusters. Interestingly, numerous factors of the ATF family and EGR-1 are themselves subject of activity-dependent transcriptional regulation, suggesting transcriptional feed-forward or feedback loops. Binding motifs recognized by AP2 were overrepresented in genes of cluster 3 with maximal induction 8 h after onset of seizure. AP1, a family of homo- and heterodimers of factors such as the transcriptionally induced cFos, Fra1 and Jun, and a heterogeneous group of additional factors may control expression of genes of cluster 5 that are maximally induced at 24 hours. We found that the promoters of the genes of cluster 5 are significantly depleted of CpG-dinucleotides, suggesting that these genes may not be epigenetically controlled.

**Table 2:** Overrepresentation of conserved transcription factor binding sites in genes of indicated clusters.

Cluster	Transcripton factor binding sites conserved between mouse and human	Transcripton factor binding sites only significant in mouse
1	CREB/ATF	TBX5
2	CREB/ATF	SP1, EGR-1
3	AP2	none
4	none	none
5	PU1, NFkB, c-Ets-2, HNF1	ICSBP, IRF, AP1, STAT, NRF2

* AP1, complex of transcription factors FOS and JUN; AP2, transcription factor AP-2; ATF, activating transcription factor; CREB, cAMP responsive element binding protein 1; c-Ets-2, v-ets erythroblastosis virus E26 oncogene homolog 2; EGR-1, Early Growth Response 1 (KROX family of transcription factors); HNF1, HNF1 homeobox A; ICSBP, interferon consensus sequence binding protein/interferon regulatory factor 8; IRF, interferon regulatory factor 1; SP1, trans-acting transcription factor 1; NFkB, nuclear factor kappa B; NRF2, NF-E2-related factor-2; PU1, Transcription factor PU.1/Sfpi1; STAT, signal transducer and activator of transcription; TBX5, T-box transcription factor 5.

## Discussion

Changes in gene expression are central for translating synaptic activity into sustained changes of synaptic strength. We used genome-wide microarray profiling to analyze changes in gene expression at 4 different time points after the induction of synaptic activity and assigned genes to five clusters with different transcriptional profiles. Many of the identified activity-induced genes have been previously implicated in morphogenesis, cytoskeleton remodeling and synapse formation and presumably play a role in synaptic and axonal remodeling observed in the hippocampus following kainic acid induced seizures [36]. In contrast, cell death has been frequently studied and observed after longer time periods, usually days after seizures [17]. Moreover, C57BL/6 mice show minimal cell death in hippocampus and other brain regions following kainic acid induced seizures [17]. In keeping with these observations, our functional annotation of activity-induced genes within the first 24 h after seizure onset revealed only a small number of genes that can be related to apoptosis or stress response. Moreover, no specialized biological function was overrepresented in any of the clusters.

The microarray data of our study is of high reliability because (i) a large number of already known activity-induced genes were identified and (ii) induction of all arbitrarily tested genes of the microarray analysis could be confirmed in independent experiments using *in situ* hybridizations. Radioactive *in situ* hybridizations validate quantitative changes in expression and these analyses provide additional information on the localization of expression of the specific genes in the brain. For example expression of Arfl4 is confined to the dentate gyrus, expression of other genes, such as GPR19 and Arl5b, are induced in the dentate gyrus and in CA1, whereas GPR115 expression is elevated in CA3 and the dentate gyrus. Besides hippocampus, we also found induced gene expression levels in other brain regions. An example can be seen in the expression of Rras2. Transcripts are maximally induced 2 h after seizure in the CA1 and dentate gyrus, while at 4 h maximal expression is observed in the cerebral cortex. In conclusion, our studies demonstrate that activity-regulated genes are induced with distinct kinetics in different areas of the hippocampus.

The transcriptional induction of genes is a central mechanism that allows neurons to respond to specific stimuli. Activity-regulated genes can be divided into two classes: (i) immediate early genes (IEGs) whose transcription is activated rapidly and is independent of protein synthesis and (ii) late response genes whose transcription is induced more slowly and is dependent on new protein synthesis [6]. Many IEGs have been shown to encode transcription factors directing specific programs of late gene expression and orchestrating long-term responses [6]. Our analysis of promotor regions of co-regulated genes identifies binding sites of transcription factors that are themselves subject to activity-dependent transcriptional regulation. This suggests feed-forward or feedback events regulating the level of later responding genes. Thus the majority of the late induced genes of cluster 3–5 are expected to be non-IEGs that are regulated secondary to seizure effects on IEG transcription factors.

Activity-regulated gene transcription can be further modulated by regulation of mRNA splicing. Two categories of alternative splicing are thought to play a major role in synaptic remodeling, (i) alternative usage of 3′-UTRs resulting in the generation of mRNAs harboring different cis-regulatory elements, but encoding identical proteins and (ii) alternative exon usage resulting in the expression of alternative proteins which can interfere with or alter the function of preexisting proteins.

The 3′-UTRs of mRNAs can contain sequences that influence their stability, subcellular localization and translation [37]. Alternative usage of 3′-UTRs allows differential targeting of mRNAs to subcellular compartments and a posttranscriptional control of mRNA translation and stability. We identified an activity-regulated variant of Zwint which harbors an alternatively spliced 3′-UTR. Zwint may play a role in presynaptic membrane trafficking [38], however, a dendritic localization of Zwint has also been reported [39], [40]. We here demonstrate that Zwint is alternatively spliced. Splice-variant 1 exhibits a very low basal expression in neurons, but is substantially induced by synaptic activity in granule cells of the dentate gyrus. In contrast, we find that splice-variant 2 is constitutively expressed in many areas of the brain, including the dentate gyrus. The splice variants represent an example of 3′exon switching and differ in their terminal exons which correspond to mutually exclusive 3′-UTRs. Only the activity-regulated 3′-UTR of variant 1 harbors two putative activity-dependent polyadenylation signals [34] and multiple cis-acting elements implicated in the regulation of mRNA stability and transport [31], [32], [33]. These include K-boxes and U-rich motifs, which are conserved miRNA target sites in 3′-UTRs and mediate negative post-transcriptional regulation [32], [33]. Therefore, the activity-induced Zwint transcript may be regulated by miRNA-dependent mechanisms. The alternative usage of two mutually exclusive 3′-UTRs of Zwint is an example for an activity induced switch from a constitutive 3′-UTR lacking regulatory elements to a 3′-UTR with multiple cis-acting control elements. These results suggest that in the wake of activity there may be changes in the stability, efficiency of translation, and perhaps in trafficking of RNA transcripts.

Alternative exon usage can result in the expression of alternative proteins. The generation of an alternative protein may result in a dominant negative functional block of the constitutively expressed variant that may destabilize existing synaptic structures. A well-studied example for such activity-regulated alternative splicing is Homer1 [41]. We here identified activity-regulated splice variants of Erc1 and Erc2 that similarly might have dominant negative functions. These activity-regulated splice variants encode alternative proteins that are likely to be functionally distinct of the constitutively expressed variants. Interestingly, like Homer1, Erc1 and Erc2 are scaffold proteins. However, unlike Homer1 they are enriched in the presynaptic active zone [42]. This is in keeping with our finding that a number of genes encoding components of the presynaptic compartment including Tomosyn/Stxbp5 and Rim4, are induced by synaptic activity. We currently do not know whether activity induced expression is pre- or postsynaptic. Future studies will also be required to see if the activity-induced splice variants of Erc1 and 2 might serve dominant negative functions. The expression of a dominant negative variant of a protein may be one important mechanism to acutely block the function of a protein that inhibits plastic changes. Another regulatory mechanism may be adjusting expression levels of proteins which negatively regulate plasticity. It has been suggested that plasticity requires not only the activation of positive regulatory mechanisms but also the removal of inhibitory constrains [43]. In this view plasticity is mediated not only by positive but also negative regulatory mechanisms, in much the same way as cell division is controlled by the proteins encoded by oncogenes and tumor suppressor genes [44]. Thus, down-regulation of gene expression by neuronal activity may be an important mechanism by which neuronal plasticity modulates synapses, but information on plasticity suppressor genes is still scarce.

In this study we noticed a particular unusual expression kinetic for Rasl10a. Complex expression profiles in response to seizures have been observed before. One example can be seen in expression of nerve growth factor (NGF) after hilus lesion-induced seizures. NGF expression follows a biphasic profile with increased expression after 4 h followed by a strong decrease and another increase of expression after 24 h [45]. Another example of complex biphasic induction after seizure activity has been described for dynorphin although over a period of days [46], [47]. So far, the small GTPase Rasl10a is poorly characterized. Rasl10a is exclusively expressed in neuronal tissue [45]. Moreover, it has been demonstrated that most neuronal tumor cell lines lose Rasl10a expression and that Rasl10a has a tumor suppressor potential [46], [47]. Here we show that transcript levels are dramatically decreased in the hippocampus and the cerebral cortex 1 and 2 h after seizure onset. However, after 4 h Rasl10a levels return to baseline and are highly induced after 8 h. This dynamic expression pattern suggests that Rasl10a might have a function in the stabilization of synaptic structures and needs to be downregulated in order for plastic changes to take place. In this respect Rasl10a might function as suppressor of plasticity.

## Supporting Information

Figure S1 Densiometric quantification of *in situ* hybridizations of examples of cluster 1 (A) and 2 (B). Quantification of autoradiograms of coronal sections of mouse brains at different time points following seizure. Radioactive *in situ* hybridizations of sections were conducted in parallel on one glass slide using gene specific antisense RNA probes and detected by autoradiography. The relative staining intensity in the hippocampal formation was quantified and normalized to the 0 h time point. Error bars represent SEM (n = 2). One series of autoradiograms is shown below the respective quantification.
(TIF)

Figure S2 Quantification of *in situ* hybridizations of examples of cluster 3 (A), 4 (B), and 5 (C). Quantification of autoradiograms of coronal sections of mouse brains at different time points following seizure. Radioactive *in situ* hybridizations of sections were conducted in parallel on one glass slide using gene specific antisense RNA probes and detected by autoradiography. The relative staining intensity in the hippocampal formation was quantified and normalized to the 0 h time point. Error bars represent SEM (n = 2). One series of autoradiograms is shown below the respective quantification.
(TIF)

Table S1 Identified neuronal activity-regulated genes. Assignments to clusters are indicated in Roman numbers. The number of probe sets in each cluster is indicated in Arabic numbers. If neuronal activity-induced expression was previously reported, the publication reference is given.
(PDF)
