# Supplementary figures and images for: Genome-Wide Profiling of the Activity-Dependent Hippocampal Transcriptome
Source: PLoS One. 2013 Oct 17;8(10):e76903. doi: 10.1371/journal.pone.0076903 (PMC3798291; doi:10.1371/journal.pone.0076903)

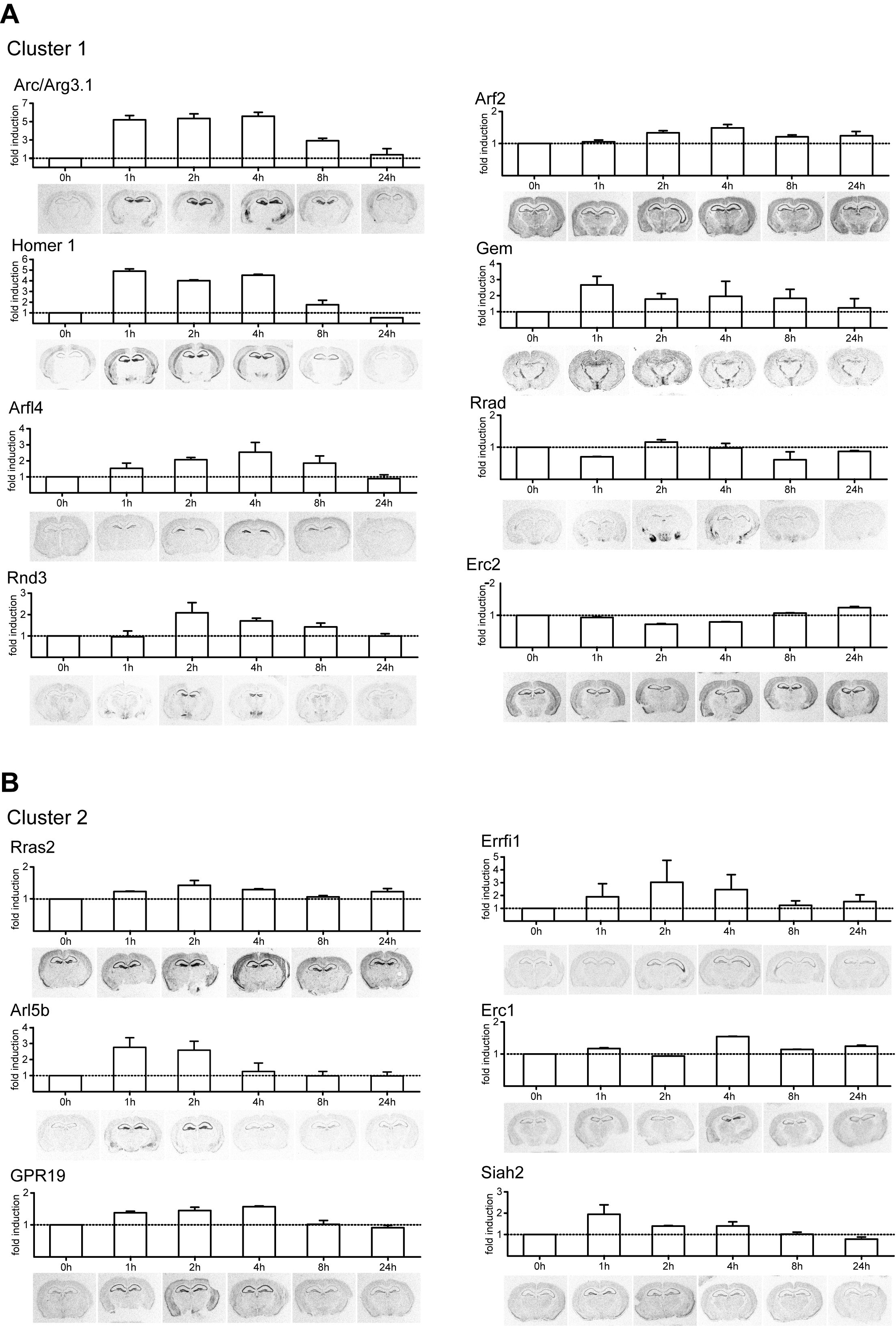

Supplement: Figure S1 — Densiometric quantification of in situ hybridizations of examples of cluster 1 (A) and 2 (B). Quantification of autoradiograms of coronal sections of mouse brains at different time points following seizure. Radioactive in situ hybridizations of sections were conducted in parallel on one glass slide using gene specific antisense RNA probes and detected by autoradiography. The relative staining intensity in the hippocampal formation was quantified and normalized to the 0 h time point. Error bars represent SEM (n = 2). One series of autoradiograms is shown below the respective quantification. (TIF) [file pone.0076903.s001.tif]

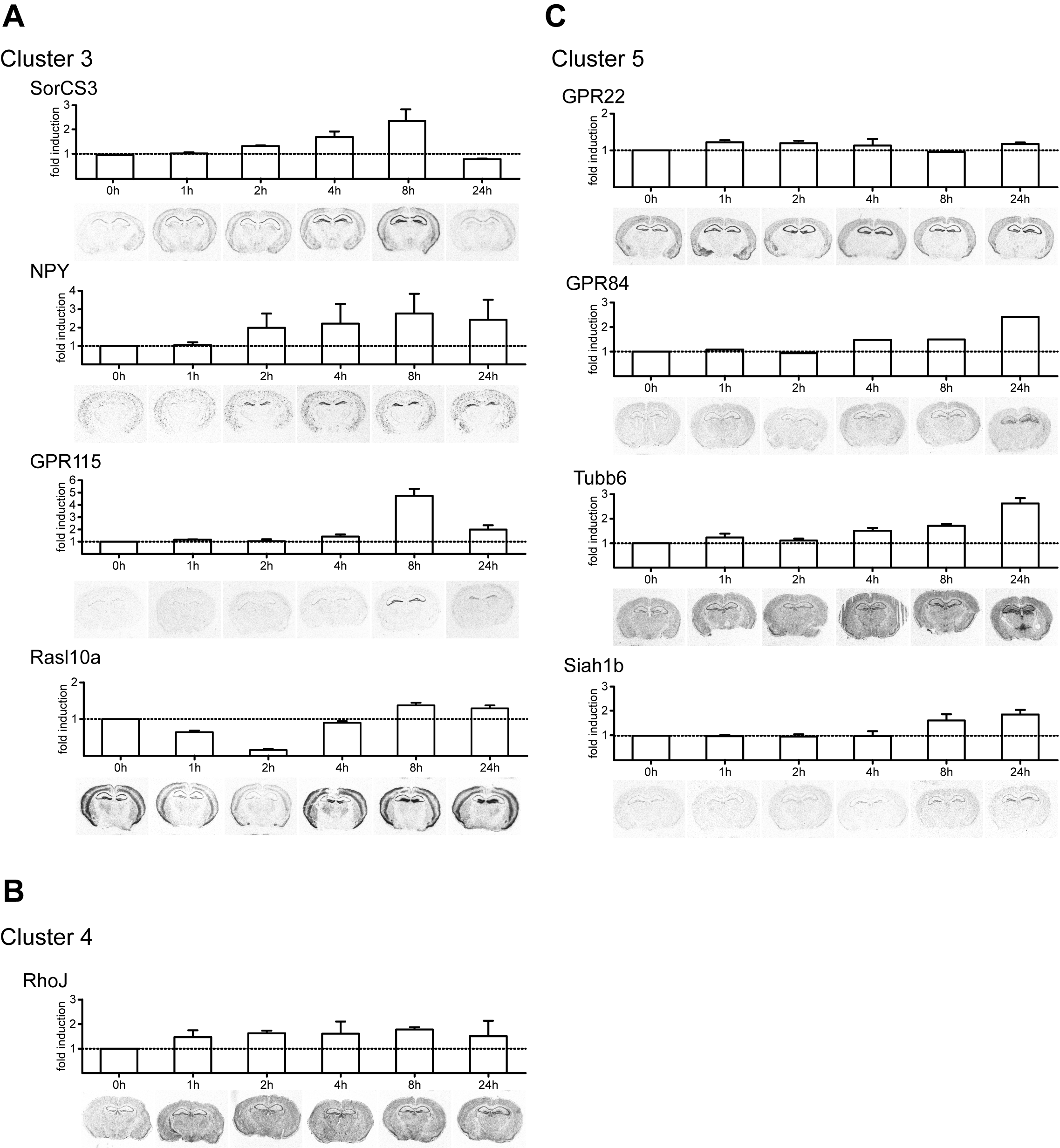

Supplement: Figure S2 — Quantification of in situ hybridizations of examples of cluster 3 (A), 4 (B), and 5 (C). Quantification of autoradiograms of coronal sections of mouse brains at different time points following seizure. Radioactive in situ hybridizations of sections were conducted in parallel on one glass slide using gene specific antisense RNA probes and detected by autoradiography. The relative staining intensity in the hippocampal formation was quantified and normalized to the 0 h time point. Error bars represent SEM (n = 2). One series of autoradiograms is shown below the respective quantification. (TIF) [file pone.0076903.s002.tif]
