# Supplementary material for: Genome-Wide Profiling of the Activity-Dependent Hippocampal Transcriptome
Source: PLoS One. 2013 Oct 17;8(10):e76903. doi: 10.1371/journal.pone.0076903 (PMC3798291; doi:10.1371/journal.pone.0076903)
Supplement: Table S1 — Identified neuronal activity-regulated genes. Assignments to clusters are indicated in Roman numbers. The number of probe sets in each cluster is indicated in Arabic numbers. If neuronal activity-induced expression was previously reported, the publication reference is given. (PDF) [file pone.0076903.s003.pdf]

Gene name or Genbank Accession number	Cluster					Gene/Protein	Reference
	I	II	III	IV	V		
0610010D24Rik	1	0	0	0	0	Ccdc88c	
0910001A06Rik	0	0	0	0	1		
1110003E01Rik	1	0	1	0	0		
1110017I16Rik	0	0	0	0	1		
1190002N15Rik	0	2	0	0	0		
1200016E24Rik	2	0	0	0	0		
1300007C21Rik	0	0	0	2	0		
1600029D21Rik	1	0	0	0	0		
1700010C24Rik	0	0	1	0	0		
1700020I14Rik	0	0	0	0	1		
1700054N08Rik	0	0	0	2	0		
1700086L19Rik	0	1	0	0	0		
1810011O10Rik	0	2	0	0	0		
1810013L24Rik	0	0	0	1	0		
1810029B16Rik	1	1	0	0	0		
2010002N04Rik	1	0	0	0	0		
2010109K11Rik	0	0	0	0	1		
2010111I01Rik	1	0	0	0	0		
2010200O16Rik	0	0	0	1	0		
2010310D06Rik	1	0	0	0	0		
2210403K04Rik	1	0	0	0	0		
2300002D11Rik	0	0	1	0	0		
2310008H04Rik	0	0	0	0	1		
2310043N10Rik	0	1	0	0	0		
2410025L10Rik	0	0	0	1	0		
2410042D21Rik	0	0	0	0	1		
2610301G19Rik	0	0	0	1	0		
2610528G24Rik	1	0	0	0	0		
2900011L18Rik	0	1	0	0	0		
2900064F13Rik	0	0	1	0	0		
2900097C17Rik	0	0	0	1	0	Bscl2, Bernardinelli-Seip congenital lipodystrophy 2 homolog	
3021401C12Rik	1	0	0	0	0		
3110001A13Rik	0	3	0	0	0		
3300001A09Rik	0	0	1	0	0		
3830612M24	0	0	1	0	0		
4632417K18Rik	0	0	0	0	1		
4930430F08Rik	0	0	0	0	1		
4930431B11Rik	0	0	0	0	1		
4930434J08Rik	1	0	0	0	0		
4933407C03Rik	0	0	1	0	0		
5031439G07Rik	0	0	1	0	0		
5033430I15Rik	1	0	0	0	0		
5330406M23Rik	0	1	0	0	0		
5730406M06Rik	0	0	0	1	0	serine/arginine-rich splicing factor 18, Sfrs18	
5730557B15Rik	2	0	0	0	0		
5730601F06Rik	0	0	0	1	0		

5830411G16Rik	0	0	0	1	0		
6030405A18Rik	1	0	0	0	0		
6330403K07Rik	0	0	0	0	1		
6330406I15Rik	0	0	0	0	2		
6330416L11Rik	1	0	0	0	0		
6330418B08Rik	0	0	1	0	0		
6720475J19Rik	0	0	1	0	0		
8430415E04Rik	0	0	1	0	0	Ppp4r4, protein phosphatase 4, regulatory subunit 4	
9030624J02Rik	0	0	0	0	1		
9130211I03Rik	0	0	1	0	0		
9130404D14Rik	0	0	1	0	0		
9430012M22Rik	0	1	0	0	0		
9430029L20Rik	0	0	0	1	0		
A030009H04Rik	0	0	0	1	0		
A130040M12Rik	1	0	2	0	0		
A2m	0	0	0	0	1	alpha-2-macroglobulin	
A530050D06Rik	0	0	0	0	1		
A630082K20Rik	0	0	0	1	0		
AA930108	0	1	0	0	0		
AA987161	0	0	0	0	1		
AI256396	0	0	1	0	0		
AI315324	0	0	1	0	0		
AI451617	0	0	0	0	1		
AI503301	0	0	0	1	0		
AI646383	0	1	0	0	0		
AI788669	0	0	0	1	0	Arf6	
AI844718	0	1	0	0	0		
AI848100	0	1	0	1	0		
AU067731	1	0	0	0	0		
AW112037	0	0	0	1	0		
AW456874	1	1	0	0	0	expressed sequence AW456874	
Aasdhppt	0	0	0	0	1	aminoadipate-semialdehyde dehydrogenase- phosphopantetheinyl transferase	
Abcb7	0	0	0	1	0	ATP-binding cassette, sub-family B (MDR/TAP), member 7	
Abcd2	0	0	0	1	0	Tap1 transporter 1, ATP-binding cassette, sub-family B	
Abi3bp	0	0	0	0	1	Abi3bp ABI gene family, member 3 (NESH) binding protein	
Abr	1	0	0	0	0	active BCR-related gene	
Acbd3	0	0	0	0	1	Golgi resident protein GCP60; acyl-Coenzyme A binding domain containing 3	
Acly	0	0	0	1	0	ATP citrate lyase	
Acsl4	0	0	1	2	0	acyl-CoA synthetase long-chain family member 4	
Actn1	0	1	0	0	0	actinin, alpha 1	
Actr2	0	0	0	1	0	ARP2 actin-related protein 2 homolog	
Acvr1	0	0	1	1	0	activin A receptor, type 1; BMP receptor	
Acvr1c	0	0	1	0	0	Acvr1c activin A receptor, type IC	
Ada	0	1	0	0	0	adenosine deaminase	
Adam10	0	0	0	0	2	a disintegrin and metallopeptidase domain 10	
Adamts1	0	0	0	1	0	ADAM metallopeptidase with thrombospondin type 1 motif, 1	
Adamts9	0	1	0	0	0	ADAM metallopeptidase with thrombospondin type 9 motif, 9	
Adck4	0	0	0	0	1	aarF domain containing kinase 4	
Adm	0	0	0	1	0	adrenomedullin	
Adra1a	0	0	1	0	0	adrenergic receptor, alpha 1a	
Adra2c	0	0	1	0	0	adrenergic receptor, alpha 2c	
Aff1	1	0	0	0	0	AF4/FMR2 family, member 1	
Aff3	0	1	0	0	0	AF4/FMR2 family, member 3	

Agc1	0	1	0	0	0	aggrecan 1	
Agpat7	0	1	0	0	0	lysophosphatidylcholine acyltransferase 4	
Agtpbp1	0	0	0	1	0	ATP/GTP binding protein 1;cytosolic carboxypeptidase 1	
Aim1l	0	0	1	0	0	absent in melanoma 1-like	
Akap12	1	0	0	0	0	A kinase (PRKA) anchor protein (gravin) 12	
Akap13	0	1	0	0	0	A kinase (PRKA) anchor protein 13	
Akr1b8	0	0	0	0	1	aldo-keto reductase family 1, member B8	
Aldh1l1	0	0	0	1	0	aldehyde dehydrogenase 1 family, member L1	
Aldh1l2	0	0	0	0	1	aldehyde dehydrogenase 1 family, member L2	
Alg13	1	0	0	0	0	asparagine-linked glycosylation 13 homolog	
Ampd3	0	0	0	0	1	adenosine monophosphate deaminase 3	
Ankrd28	0	0	0	0	1	ankyrin repeat domain 28	
Anks1b	0	0	0	1	0	ankyrin repeat and sterile alpha motif domain containing 1B	
Anxa2	0	0	1	0	0	annexin A2	
Anxa3	0	0	0	0	1	annexin A3	
Anxa5	0	0	0	0	1	annexin A5	
Ap2a2	0	0	0	2	0	adaptor protein complex AP-2, alpha 2 subunit	
Ap2b1	1	0	0	0	0	adaptor-related protein complex 2, beta 1 subunit	
Ap2m1	0	0	0	1	0	adaptor protein complex AP-2, mu1	
Apold1	0	1	0	0	0	apolipoprotein L domain containing 1, Verge (vascular early response gene)	1
App	0	0	0	1	0	amyloid beta (A4) precursor protein	2,3
Aqp4	0	0	0	0	3	aquaporin 4	
Arc/Arg3.1	1	0	0	0	0	activity regulated cytoskeletal-associated protein, arg3.1	4,5
Arf2	2	0	0	0	0	ADP-ribosylation factor 2	
Arfl4	1	0	0	0	0	ADP-ribosylation factor 4-like	
Arhgap17	0	0	1	0	0	Rho GTPase activating protein 17, nadrin	
Arhgef7	0	0	1	0	0	Rho guanine nucleotide exchange factor (GEF7), PAK-interacting exchange factor beta	
Arid5a	1	0	0	0	0	AT rich interactive domain 5A (Mrf1 like)	
Arid5b	1	1	0	0	0	AT rich interactive domain 5B (Mrf1 like)	
Arih1	0	0	0	1	0	ariadne ubiquitin-conjugating enzyme E2 binding protein homolog 1	
Arih2	1	0	0	0	0	ariadne homolog 2, TRIAD1	
Arl5a	0	0	0	1	0	ADP-ribosylation factor-like 5A	
Arl5b	1	1	0	0	0	ADP-ribosylation-like factor 8, 5B	
Arl6ip2	0	0	0	0	1	ADP-ribosylation factor-like 6 interacting protein 2, Aip-2	
Arl6ip5	1	0	0	0	0	ADP-ribosylation factor-like 6 interacting protein 5, Aip-5, Gtrap3-18	
Arl8b	0	0	0	1	0	ADP-ribosylation factor-like 8B	
Armcx3	1	0	0	0	0	armadillo repeat containing, X-linked 3	
Arpc1b	0	0	0	0	1	actin related protein 2/3 complex, subunit 1B	
Arpc4	0	0	0	1	0	actin related protein 2/3 complex, subunit 4	
Arpp19	2	0	0	0	0	cAMP-regulated phosphoprotein 19	
Arpp21	2	0	0	0	0	cyclic AMP-regulated phosphoprotein	
Arrdc4	0	0	0	0	1	arrestin domain containing 4	
Arsj	0	0	0	0	1	arylsulfatase J	
Arts1	0	0	0	0	1	Erap1 = endoplasmic reticulum aminopeptidase 1	
Asb11	1	0	0	0	0	ankyrin repeat and SOCS box-containing protein 11	
Asph	1	0	0	0	0	aspartate-beta-hydroxylase	
Atf2	0	0	0	1	0	activating transcription factor 2, cAMP responsive element binding protein 2,Creb2	
Atf3	1	0	0	0	0	ATF/CREB; activating transcription factor 3	
Atf4	1	0	1	0	0	activating transcription factor 4, C/ATF, CREB2	
Atp11c	0	1	1	0	0	Atpase, class VI, type 11C	
Atp13a4	0	0	0	0	1	ATPase type 13A4	
Atp1a1	2	0	0	0	0	ATPase, Na+/K+ transporting, alpha 1 polypeptide	
Atp1a3	0	0	0	1	0	ATPase, Na+/K+ transporting, alpha 3 polypeptide	

Atp1b1	0	0	0	1	0	ATPase, Na+/K+ transporting, beta 1 polypeptide	
Atp2c1	0	0	0	2	0	ATPase, Ca++-sequestering	
Atp6v0a1	0	0	0	2	0	ATPase, H+ transporting, lysosomal V0 subunit A1	
Atrx	0	0	0	1	0	alpha thalassemia/mental retardation syndrome X-linked homolog	
Auh	0	2	0	0	0	AU RNA binding protein/enoyl-coenzyme A hydratase	
Axud1	1	0	0	0	0	AXIN1 up-regulated 1; TGF-beta induced apoptosis protein 3	
Azin1	0	0	0	2	0	antizyme inhibitor 1, Oazin	
B230216N24Rik	1	0	0	0	0	hypothetical protein LOC78603	
B3gnt2	0	0	0	1	0	UDP-GlcNAc:betaGal beta-1,3-N-acetylglucosaminyltransferase 2	
B3gnt5	0	0	0	0	1	UDP-GlcNAc:betaGal beta-1,3-N-acetylglucosaminyltransferase 5	
BB220380	0	0	1	0	0	Pik3r6 = phosphoinositide-3-kinase, regulatory subunit 6	
BC004022	0	0	1	0	0	LOC668085	
BC011467	1	0	0	0	0		
BC016423	0	0	0	0	1	PBR associated protein; peripherial benzodiazepine receptor associated protein	
BC018242	0	0	1	0	0	lipid phosphate phosphatase-related protein type 2	
BC022687	0	0	1	0	0	hypothetical protein LOC217887	
BC032204	0	0	0	0	2	UNC-112 related protein 2, Kindlin-3, actin-associated	
BC033915	0	2	0	0	0	hypothetical protein LOC70661	
BC049806	0	0	2	0	0	hypothetical protein LOC213056	
BC049807	1	0	0	0	0	hypothetical protein LOC381066	
BC063749	0	1	0	0	0	DANGER, KIAA1754, mKIAA1754	
BC065085	1	0	0	0	0		
BC067047	0	0	1	0	0	Setd6; P-REX1	
BF642829	0	0	0	0	2	Smit1, Slc5a3, solute carrier family 5 (inositol transporters), member 3	6
Baalc	0	0	3	0	0	brain and acute leukemia, cytoplasmic	
Bach1	0	0	1	0	0	BTB and CNC homology 1, basic leucine zipper transcription factor 1	
Bag3	1	0	0	0	0	Bcl2-associated athanogene 3; Bcl-2-interacting death supressor	
Baiap2	0	3	0	0	0	brain-specific angiogenesis inhibitor 1-associated protein 2	
Baz1a	2	0	0	0	0	bromodomain adjacent to zinc finger domain,1A; ATP-dependent chromatin remodeling protein	
Bcl3	0	0	0	0	1	B-cell leukemia/lymphoma 3	
Bcl6	0	1	0	0	0	B-cell leukemia/lymphoma 6	
Bdnf	2	0	0	0	0	Brain-derived neurotrophic factor	7-9,10
Bgn	0	0	0	0	2	biglycan, DSPG1, PG-S1, PGI, SLRR1A	
Blnk	0	0	1	0	0	B-cell linker protein; lymphocyte antigen 57	
Blvrb	1	0	0	0	0	biliverdin reductase B	
Bpnt1	1	0	0	0	0	bisphosphate 3'-nucleotidase 1	
Braf	1	0	0	0	0	Braf transforming gene, B-Raf proto-oncogene serine/threonine-protein kinase (p94)	
Brcc3	0	0	0	1	0	Brcc3 (BRCA1/BRCA2-containing complex, subunit 3)	
Btbd3	0	0	0	1	0	BTB (POZ) domain containing 3	
Btg2	0	2	0	0	0	B-cell translocation gene 2; NGF-inducible anti-proliferative protein PC3	11,12
Btg4	0	1	0	0	0	B-cell translocation gene 4 , PC3B	
Bzw1	0	0	0	4	0	basic leucine zipper and W2 domains 1	
C030009J22Rik	0	0	1	0	0		
C030018P15Rik	0	1	0	0	0		
C030019I05Rik	1	0	0	0	0	MGC27121	
C030046I01Rik	0	2	0	0	0	hypothetical protein LOC109284	
C130006E23	0	0	1	0	0	hypothetical protein C130006E23	
C130065N10Rik	0	0	2	0	0	hypothetical protein LOC213056, D1Ertd53e	
C1qb	0	0	0	0	3	complement component 1, q subcomponent, beta polypeptide	13
C330006A16Rik	0	1	0	0	0		
C4b	0	0	0	0	1	complement component 4B	13
C530044C16Rik	0	0	0	0	1		

C79445	0	0	0	0	1	Tubb2a-ps2 = tubulin, beta 2a	
C85699	0	1	0	0	0		
Cacna2d1	0	0	0	1	0	Cacna2d1 calcium channel, voltage-dependent, alpha2/delta subunit 1	
Cacng8	0	1	0	0	0	alcium channel, voltage-dependent, gamma subunit 8	
Camk2d	0	0	0	0	1	calcium/calmodulin-dependent protein kinase II, delta	
Canx	0	0	0	2	0	calnexin	
Cap1	0	1	0	0	0	CAP, adenylate cyclase-associated protein 1	
Capg	0	0	0	0	1	capping protein, gelsolin-like, gCap39, mbh1	
Car10	0	0	1	0	0	carbonic anhydrase 10	
Car12	0	0	1	0	0	carbonic anyhydrase 12	
Car8	0	0	0	0	1	carbonic anhydrase 8	
Cav1	0	0	0	0	1	caveolin, caveolae protein 1	14
Cav2	0	0	0	0	1	caveolin 2	
Cbln1	0	0	0	0	1	cerebellin 1 precursor protein	
Ccdc109a	0	2	0	0	0	coiled-coil domain containing 109A	
Ccdc49	0	1	0	0	0	coiled-coil domain containing 49	
Ccdc80	0	0	0	0	1	coiled-coil domain containing 80, steroid sensitive gene 1; Urb	
Ccl12	0	0	0	0	1	chemokine (C-C motif) ligand 12	
Ccl2	0	0	0	0	1	chemokine ligand 2, MCP-1	15
Ccl3	0	0	1	0	0	hemokine ligand 3, macrophage inflammatory protein-1alpha	
Ccnc	0	0	0	0	1	cyclin C	
Ccnf	0	0	1	0	0	cyclin F, FBXO1	
Ccnt2	0	0	1	0	0	cyclin T2	
Ccr5	0	0	0	0	2	chemokine receptor 5, GPCR	
Ccrn4l	1	0	0	0	0	CCR4 carbon catabolite repression 4-like	
Cd14	1	0	0	0	0	CD14 antigen	
Cd151	0	0	0	0	1	SFA-1; PETA-3; Tspan24	
Cd1d1	0	0	2	0	0	CD1d1 antigen	
Cd44	0	0	0	0	3	HERMES, hyaluronan receptor CD44	16
Cd68	0	0	0	1	0	Scard1, gp110; macrosialin	
Cd9	0	0	0	0	1	CD9 antigen; Tspan29	
Cd93	0	0	2	0	0	CD93 antigen;	
Cda	0	0	1	0	0	cytidine deaminase	
Cdc27	0	0	0	1	0	cell division cycle 27 homolog	
Cdc2l1	1	0	0	0	0	cell division cycle 2-like 1	
Cdh2	0	0	0	1	0	cadherin 2; N-cadherin, Ncad	
Cdkn1a	2	0	0	0	0	cyclin-dependent kinase inhibitor 1A	
Cdo1	0	0	0	1	0	cysteine dioxygenase, type I	
Cds2	0	0	0	0	1	CDP-diacylglycerol synthase 2	
Cdt1	0	0	0	1	0	chromatin licensing and DNA replication factor 1	
Cdyl	0	0	2	0	0	chromodomain protein	
Cebpb	2	0	0	0	0	CCAAT/enhancer binding protein (C/EBP), beta; NF-IL6, TCF5	
Cebpd	0	0	0	1	0	Aplysia, required for memory concolidation	
Cenpa	1	0	0	0	0	centromere protein A; Cenp-A	
Cfl1	0	1	0	0	0	Cofilin1	
Cflar	0	0	4	0	0	CASP8 and FADD-like apoptosis regulator	
Cggbp1	0	0	0	1	0	CGG triplet repeat binding protein 1	
Cgref1	0	0	2	0	0	cell growth regulator with EF hand domain 1	
Chfr	0	0	0	1	0	checkpoint with forkhead and ring finger domains	
Chga	0	0	1	0	0	chromogranin A	17
Chmp1b	0	0	1	0	0	chromatin modifying protein 1B	
Chst11	0	0	2	0	0	carbohydrate sulfotransferase 11	
Chst9	0	0	0	0	1	carbohydrate sulfotransferase 9	

Cirbp	0	1	0	0	0	cold inducible RNA binding protein	
Cited2	0	1	0	0	0	Cbp/p300-interacting transactivator 2	
Ckb	0	1	0	0	0	creatine kinase, brain	
Clcf1	0	0	0	0	2	cardiotrophin-like cytokine factor 1	
Clic1	0	0	0	0	1	chloride intracellular channel 1	
Clint1	0	0	0	0	1	clathrin interactor 1	
Clstn3	0	1	0	0	0	calsyntenin 3	
Cltb	0	0	1	1	0	clathrin, light polypeptide (Lcb)	
Cltc	1	2	0	0	0	clathrin, heavy polypeptide (Hc); CHC17	18
Cnn3	0	0	0	3	3	calponin 3, acidic	
Cntn3	0	1	2	0	0	contactin 3; Pang	
Col11a1	0	0	0	0	2	collagen, type XI, alpha 1	
Col16a1	0	0	0	0	1	collagen, type XVI, alpha 1	
Col4a1	1	0	0	0	0	collagen, type IV, alpha 1	
Coq10b	2	0	0	0	0	coenzyme Q10 homolog B	
Coro1c	0	0	0	1	0	coronin, actin binding protein 1C	
Cox15	0	0	1	0	0	COX15 homolog, cytochrome c oxidase assembly protein	
Cpeb2	0	0	0	2	0	cytoplasmic polyadenylation element binding protein 2	19
Cpeb4	0	0	0	1	0	cytoplasmic polyadenylation element binding protein 4	19
Cpsf6	0	0	0	1	0	cleavage and polyadenylation specific factor 6	
Creb3l2	0	0	1	0	0	cAMP responsive element binding protein 3-like 2	
Creld2	0	0	1	0	0	cysteine-rich with EGF-like domains 2	
Crem	2	0	0	0	0	cAMP responsive element modulator	20
Crh	1	0	0	0	0	corticotropin releasing hormone	21, 22
Crhbp	0	0	1	0	0	corticotropin releasing hormone binding protein	
Crispld2	0	0	1	0	0	cysteine-rich secretory protein LCCL domain containing 2	
Crsp2	1	1	0	1	0	cofactor required for Sp1 transcriptional activation, subunit 2	
Crtac1	0	0	3	0	0	cartilage acidic protein 1	
Cry1	0	0	1	0	0	cryptochrome 1 (photolyase-like)	
Csda	0	0	0	2	0	cold shock domain protein A	
Csde1	0	0	0	1	0	cold shock domain containing E1	
Csf2rb1	0	0	0	0	1	colony stimulating factor 2 receptor, beta	
Ctgf	0	0	1	0	0	connective tissue growth factor	23
Cthrc1	0	0	0	0	1	collagen triple helix repeat containing 1	
Ctla2a	0	0	2	0	0	cytotoxic T lymphocyte-associated protein 2 alpha	
Ctla2b	0	0	1	0	0	cytotoxic T lymphocyte-associated protein 2 beta	
Ctnnd1	0	1	0	0	0	catenin (cadherin associated protein), delta 1	
Ctnnd2	0	0	1	0	0	catenin (cadherin associated protein), delta 2	
Ctsc	0	0	0	0	1	cathepsin C	
Ctsz	0	0	0	0	3	cathepsin Z	
Cugbp2	0	1	0	0	0	CUG triplet repeat, RNA binding protein 2	
Cx3cl1	0	1	0	0	0	chemokine CX3CL1/FRACTALKINE,neurotactin	
Cx3cr1	0	0	0	0	1	chemokine (C-X3-C) receptor 1	
Cxcl1	1	0	0	0	0	chemokine (C-X-C motif) ligand 1	
Cxcl10	0	0	0	0	1	chemokine (C-X-C motif) ligand 10	
Cxcr4	0	0	1	0	0	chemokine (C-X-C motif) receptor 4	
Cyb5b	0	0	0	0	1	cytochrome b5 type B	
Cyb5r3	0	0	0	0	1	cytochrome b5 reductase 3	
Cyfip2	1	0	0	0	0	cytoplasmic FMR1 interacting protein 2	
Cyp1b1	0	0	1	0	0	cytochrome P450, family 1, subfamily b	
Cyp51	1	0	0	0	0	cytochrome P450, family 51	
Cyr61	2	0	0	0	0	cysteine-rich, angiogenic inducer, 61	
D10Ertd709e	0	1	0	0	0		

D12Ertd553e	0	0	0	0	2	hypothetical protein LOC76820	
D13Ertd787e	0	2	0	0	0		
D630004K10Rik	0	0	0	0	1		
Dap	0	0	0	0	1	death-associated protein	
Dars	0	0	0	1	0	aspartyl-tRNA synthetase	
Dbc1	1	0	0	0	0	deleted in bladder cancer 1	
Dcamkl1	4	0	2	2	0	double cortin and calcium/calmodulin-dependent protein kinase-like 1; Cpg16	24
Dck	0	0	0	0	1	deoxycytidine kinase	
Dcun1d1	0	0	0	1	0	DCN1, defective in cullin neddylation 1	
Ddi2	0	0	1	0	0	DNA-damage inducible protein 2	
Ddost	0	0	0	1	0	dolichyl-di-phosphooligosaccharide-protein glycotransferase	
Ddx21	0	0	0	1	0	DEAD (Asp-Glu-Ala-Asp) box polypeptide 21	
Ddx5	2	0	0	0	0	DEAD (Asp-Glu-Ala-Asp) box polypeptide 5	
Deb1	0	1	0	0	0	differentially expressed in B16F10 1	
Decr2	0	0	0	1	0	2-4-dienoyl-Coenzyme A reductase 2	
Dennd2a	0	0	0	0	1	DENN/MADD domain containing 2A	
Dhrs1	0	0	0	0	1	dehydrogenase/reductase (SDR family) member 1	
Dio2	1	2	0	0	0	deiodinase, iodothyronine, type II	
Dip2c	0	2	0	0	0	DIP2 disco-interacting protein 2 homolog C	
Dlg4	0	2	0	0	0	postsynaptic density protein 95; synapse-associated protein SAP90	
Dlgap2	0	0	1	0	0	discs, large (Drosophila) homolog-associated protein 2; Sapap2	
Dmp1	0	0	0	2	0	dentin matrix protein 1	
Dnajb5	3	0	0	0	0	DnaJ (Hsp40) homolog, subfamily B, member 5	
Dnajb6	0	0	1	1	0	DnaJ (Hsp40) homolog, subfamily B, member 6	
Dnm1	0	0	0	1	0	dynamin 1	
Dnm1l	0	0	0	0	1	dynamin 1-like	
Doc2a	0	0	0	0	1	double C2, alpha	
Dock4	0	1	0	0	0	dedicator of cytokinesis 4	
Dok5	0	0	1	0	0	docking protein 5	
Dot1l	1	0	0	0	0	DOT1-like, histone H3 methyltransferase	
Dpm3	0	0	1	0	0	dolichyl-phosphate mannosyltransferase polypeptide 3	
Dpy19l3	2	0	0	0	0	dpy-19-like 3	
Dpysl2	0	1	0	0	0	dihydropyrimidinase-like 2	
Dpysl5	0	1	0	0	0	dihydropyrimidinase-like 5; CRAM, CRMP-5	
Dr1	0	0	0	1	0	down-regulator of transcription 1	
Drd1a	0	0	1	0	0	dopamine receptor D1A	
Dscr1	1	0	0	0	0	Down syndrome critical region homolog 1; Rcan1	
Dst	0	1	0	0	0	dystonin	
Dstn	1	0	0	0	0	destrin; ADF, actin depolymerizing factor	
Dusp1	1	0	0	0	0	dual specificity phosphatase 1	25
Dusp14	1	0	0	0	0	dual specificity phosphatase 14	
Dusp4	1	0	0	0	0	dual specificity phosphatase 4	26
Dusp5	1	0	0	0	0	dual specificity phosphatase 5; cpg21	24
Dusp6	0	1	0	0	0	dual specificity phosphatase 6	26
Dyrk1a	0	0	0	2	0	dual-specificity tyrosine-phosphorylation regulated kinase 1a	
Dyrk3	0	0	1	0	0	dual-specificity tyrosine-phosphorylation regulated kinase 3	
E330037M01Rik	0	0	1	0	0		
E430014L09Rik	0	0	1	0	0		
EG666836	0	0	0	0	1		
Eaf1	0	0	1	0	0	ELL associated factor 1	
Ecm1	0	0	0	0	1	extracellular matrix protein 1	
Edg3	0	0	0	0	2	endothelial differentiation; S1pr3, sphingosine-1-phosphate receptor 3	
Edil3	0	0	0	1	0	EGF-like repeats and discoidin I-like domains 3	

Eef2k	0	0	0	1	0	eukaryotic elongation factor-2 kinase	
Efcbp1	0	0	0	0	1	EF hand calcium binding protein 1; Necab1	
Efhd2	0	0	2	0	0	EF hand domain containing 2	
Egln3	0	0	0	0	2	EGL nine homolog 3	
Egr1	0	1	0	0	0	early growth response 1; KROX-24, ZIF-268	27
Egr2	1	1	0	0	0	Krox20; early growth response 2	28
Egr3	1	0	0	0	0	early growth response 3	27
Egr4	0	1	0	0	0	early growth response 4; Egr4l1, NGFI-C	28
Eif1a	0	0	0	0	1	eukaryotic translation initiation factor 1A	
Eif2c2	0	0	1	0	0	eukaryotic translation initiation factor 2C, 2	
Eif2s3x	0	0	0	1	0	eukaryotic translation initiation factor 2, subunit 3, structural gene X-linked	
Eif4a1	0	0	0	2	0	ATP-dependent helicase; TIF4A1	
Eif4e	0	0	0	1	0	eukaryotic translation initiation factor 4E	
Eif4ebp1	0	0	0	0	1	eukaryotic translation initiation factor 4E binding protein 1	
Eif5	0	0	0	0	2	eukaryotic translation initiation factor 5	
Elavl1	0	0	0	1	0	embryonic lethal, abnormal vision	
Elk3	0	0	1	0	0	member of ETS oncogene family	
Ell2	1	0	0	0	0	elongation factor RNA polymerase II 2	
Elmo1	0	0	1	0	0	engulfment and cell motility 1	
Eltd1	1	0	0	0	0	EGF, latrophilin and seven transmembrane domain containing 1	
Emd	0	1	0	0	0	Emerin, Emery-Dreifuss muscular dystrophy	
Emp1	0	0	0	1	0	epithelial membrane protein 1, TMP	
Ensa	0	0	1	0	0	endosulfine alpha	
Epas1	0	1	0	0	0	endothelial PAS domain protein 1	
Epm2aip1	1	0	0	0	0	EPM2A (laforin) interacting protein 1	
Eprs	1	0	0	0	0	glutamyl-prolyl-tRNA synthetase	
Eps15	0	0	0	0	1	epidermal growth factor receptor pathway substrate 15	
Erbb2ip	0	0	0	0	1	Erbb2 interacting protein; Erbin	
Erc1	0	1	0	0	0	Rab6 interacting protein 2, CAST2/ERC1b	
Erc2	1	0	0	0	0	CAST1/ERC2	
Erdr1	0	0	0	0	2	erythroid differentiation regulator 1	
Errfi1	1	1	0	0	0	ERBB receptor feedback inhibitor 1; mitogen-inducible gene 6, MIG-6	
Espl1	1	0	0	0	0	extra spindle poles-like 1	
Etf1	0	0	0	1	0	eukaryotic translation termination factor 1	
Etnk1	1	0	0	1	0	ethanolamine kinase 1	
Ets1	0	0	0	1	0	E26 avian leukemia oncogene 1, 5' domain	
Etv3	3	0	0	0	0	ets variant gene 3	
Etv5	0	1	0	1	0	ets variant gene 5	
F2r	0	0	0	0	1	oagulation factor II (thrombin) receptor	
F3	0	0	0	0	1	coagulation factor III	
F8a	0	1	0	0	0	factor 8-associated gene A	
Fancd2	0	0	2	0	0	Fanconi anemia, complementation group D2	
Fbl	1	0	0	0	0	fibrillarin	
Fbxl3	0	0	0	0	1	F-box and leucine-rich repeat protein 3	
Fbxo33	1	1	0	0	0	F-box protein 33	29
Fbxo39	0	0	0	0	1	F-box protein 39	
Fcgr1	0	0	0	0	1	Fc receptor, IgG, high affinity I	
Fcgr2b	0	0	0	0	2	Fc receptor, IgG, low affinity Iib	
Fcgr3	0	0	0	0	1	Fc receptor, IgG, low affinity III	
Fem1b	1	0	1	0	0	feminization 1 homolog b	
Fgf3	0	0	1	0	0	fibroblast growth factor 3	
Fgfr1	1	1	0	0	0	fibroblast growth factor receptor 1	30
Fgfrl1	0	0	0	0	1	fibroblast growth factor receptor-like 1	

Fgl2	0	0	0	0	1	fibrinogen-like protein 2	
Flna	0	1	0	0	0	filamin, alpha	
Fmnl1	1	0	0	0	0	formin-like 1	
Fmr1	0	0	0	1	0	Frag. X mental retard. syndr. 1 homolog	
Fndc3b	0	0	1	0	0	fibronectin type III domain containing 3B	
Fos	1	0	0	0	0	Fos, c-fos	31,32
Fosb	1	0	0	0	0	FBJ osteosarcoma oncogene B	33
Fosl2	1	1	0	0	0	fos-like antigen 2; Fra-2; FRA2	31
Freq	0	1	0	0	0	frequenin, NCS-1	34,35
Frmd6	0	1	0	0	0	FERM domain containing; Willin	
Fscn1	0	0	1	1	0	fascin homolog 1	
Furin	0	0	1	0	0	furin; PC1	36
Fyttd1	0	0	0	1	0	forty-two-three domain containing 1	
G630014P10Rik	0	1	0	0	0	mastermind-like domain containing 1; Mamld1	
Gabra1	0	0	0	1	0	gamma-aminobutyric acid (GABA-A) receptor, subunit alpha 1	
Gabrb2	0	0	0	1	0	gamma-aminobutyric acid (GABA-A) receptor, subunit beta 2	
Gabrb3	0	0	0	1	0	gamma-aminobutyric acid (GABA-A) receptor, subunit beta 3	
Gadd45a	0	0	0	1	0	growth arrest and DNA-damage-inducible 45 alpha	
Gadd45b	2	0	0	0	0	growth arrest and DNA-damage-inducible, beta	18
Gadd45g	1	0	0	0	0	growth arrest and DNA-damage-inducible 45 gamma	
Gal	0	0	0	0	1	galanin	22
Galc	0	0	0	0	1	galactosylceramidase	
Galnt9	0	0	0	1	0	UDP-N-acetyl-alpha-D-galactosamine:polypeptide N-acetylgalactosaminyltransferase 9	
Galntl2	0	0	0	0	1	UDP-N-acetyl-alpha-D-galactosamine:polypeptide N-acetylgalactosaminyltransferase-like 2	
Garnl1	0	1	0	0	0	GTPase activating RANGAP domain-like	
Gatm	0	0	0	1	0	glycine amidinotransferase	
Gbp2	0	0	0	0	2	guanylate binding protein 2	
Gbp3	0	0	0	0	1	guanylate binding protein 3	
Gbp6	0	0	0	0	1	guanylate binding protein 6	
Gdpd5	0	0	1	0	0	glycerophosphodiester phosphodiesterase domain containing 5	
Gem	1	0	0	0	0	GTP binding protein (gene overexpressed in skeletal muscle)	
Gfap	0	0	0	0	3	glial fibrillary acidic protein	37
Gfra1	0	0	1	0	2	GDNFR-alpha	38
Ggta1	0	0	0	0	1	glycoprotein galactosyltransferase alpha	
Gh	0	0	0	1	0	growth hormone	
Gja1	0	1	0	0	0	gap junction protein, alpha1; connexin 43	39
Gja3	0	0	0	0	1	gap junction protein, alpha3; connexin 46	
Gjb1	0	0	0	0	1	gap junction protein, beta 1; connexin 32	39
Gla	0	0	0	1	0	galactosidase, alpha	
Glce	0	0	1	0	0	glucuronyl C5-epimerase	
Glipr2	0	0	0	0	1	GLI pathogenesis-related 2	
Glis3	0	0	0	0	1	GLIS family zinc finger 3	
Gltscr2	0	2	0	0	0	glioma tumor suppressor candidate region gene 2	
Gm131	0	0	1	0	0	gene model 131	
Gm266	0	0	0	0	1	gene model 266	
Gmeb2	1	0	0	0	0	glucocorticoid modulatory element binding protein 2	
Gmfb	0	0	0	0	3	glia maturation factor, beta	
Gmppb	1	0	0	0	0	GDP-mannose pyrophosphorylase B	
Gna12	0	1	0	0	0	guanine nucleotide binding protein, alpha 12	
Gnb1	0	1	0	0	0	guanine nucleotide binding protein, beta 1	
Gne	0	1	0	0	0	glucosamine-2-epimerase/N-acetylmannosamine kinase	
Gnptab	0	0	0	0	1	N-acetylglucosamine-1-phosphate transferase, alpha and beta subunits	
Golph3	0	0	0	1	0	golgi phosphoprotein 3	

Gpbp1	0	0	0	0	1	GC-rich promoter binding protein 1	
Gpd1	0	0	1	0	0	glycerol-3-phosphate dehydrogenase 1	
Gpnmb	0	0	0	0	1	Glycoprotein nmb; osteoactivin	
Gpr115	0	0	1	0	0	G protein-coupled receptor 115	
Gpr19	0	1	0	0	0	G protein-coupled receptor 19	
Gpr22	0	0	0	0	1	G protein-coupled receptor 22	
Gpr3	1	0	0	0	0	G-protein coupled receptor 3	
Gpr84	0	0	0	0	1	G protein-coupled receptor 84	
Gprc5a	0	1	0	0	0	G protein-coupled receptor, family C, group 5, member A	
Gprc5b	0	0	1	0	0	G protein-coupled receptor, family C, group 5, member B	
Gpt2	0	1	0	0	0	glutamic pyruvate transaminase 2	
Gramd1b	0	1	0	0	0	GRAM domain containing 1B	
Grasp	0	0	2	0	0	GRP1-associated scaffold protein; tamalin	
Grb2	0	0	1	0	0	growth factor receptor bound protein 2	
Grem2	0	0	1	0	0	gremlin 2 homolog, cysteine knot superfamily	
Gria4	0	0	0	0	1	glutamate receptor, ionotropic, AMPA4 (alpha 4); GluR-D, Glur-4	
Grik5	0	0	1	0	0	glutamate receptor, ionotropic, kainate 5 (gamma 2); KA2	
Grin1	0	1	0	0	0	glutamate receptor, ionotropic, NMDA1(zeta1); GluRdelta1, NMDAR1, NR1	40
Grin2b	0	1	0	0	0	glutamate receptor, ionotropic, N-methyl D-aspartate 2B; NMDAR2B, NR2B	40
Gsg1l	0	0	1	0	0	GSG1-like	
Gspt1	0	0	0	1	0	G1 to S phase transition 1	
Gtpbp10	0	0	0	0	1	GTP-binding protein 10 (putative)	
Gusb	0	0	0	0	1	glucuronidase, beta	
H2afz	0	0	4	0	0	H2A histone family, member Z	
H3f3a	0	0	0	1	0	H3 histone, family 3A	
Hars	1	0	1	0	0	histidyl-tRNA synthetase	
Hbegf	0	0	2	0	0	heparin-binding EGF-like growth factor	
Hbs1l	0	0	0	1	0	Hbs1-like	
Hcfc1	0	0	0	1	0	host cell factor C1	
Hck	0	0	0	0	1	hemopoietic cell kinase	
Hectd2	1	0	0	0	0	HECT domain containing 2 (FLJ37306)	
Hells	0	0	0	0	1	helicase, lymphoid specific	
Herc3	0	1	1	0	0	hect domain and RLD 3	
Herc5	0	0	0	0	1	hect domain and RLD 5	
Hif1a	0	0	0	0	1	hypoxia inducible factor 1, alpha subunit	
Hip2	0	1	0	0	0	ubiquitin-conjugating enzyme E2K;Ube2k	
Hipk2	0	1	0	0	0	homeodomain interact. protein kinase 2	
Hipk3	0	0	0	1	0	homeodomain interact. protein kinase 3	
Hk2	0	0	0	0	1	hexokinase 2	
Hmga1	0	0	0	1	0	high mobility group AT-hook 1	
Hmgb1	0	0	0	1	2	high mobility group box 1	
Hmgb2	0	1	0	1	0	high-mobility group box 2	
Hmgcr	1	0	0	0	0	3-hydroxy-3-methylglutaryl-Coenzyme A reductase; Red	
Hmgcs1	1	1	0	0	0	3-hydroxy-3-methylglutaryl-Coenzyme A synthase 1	
Hn1l	0	0	0	0	1	hematological and neurological expressed 1-like	
Hnrpa1	0	0	0	2	0	heterogeneous nucl. ribonucleoprot. A1	
Hnrpab	0	0	0	1	0	heterogeneous nucl. ribonucleoprot. A/B	
Hnrpu	0	1	0	1	0	heterogeneous nucl. ribonucleoprotein U	
Homer1	3	0	0	0	0	Homer1	41-43
Hpcal4	0	0	1	0	0	hippocalcin-like 4	
Hpgd	0	0	0	0	1	hydroxyprostaglandin dehydrogenase 15	
Hrb	0	0	0	0	1	HIV-1 Rev binding protein	
Hs3st2	0	0	2	0	0	heparan sulfate 3-O-sulfotransferase 2	

Hs6st2	0	0	0	1	0	heparan sulfate 6-O-sulfotransferase 2	
Hsd17b12	1	1	0	0	0	hydroxysteroid (17-beta) dehydrogenase 12	
Hsd17b7	0	1	0	0	0	hydroxysteroid (17-beta) dehydrogenase 7	
Hsd3b7	0	0	0	0	1	hydroxy-delta-5-steroid dehydrogenase, 3 beta- and steroid delta-isomerase 7	
Hsp110	0	0	1	1	0	heat shock 105kDa/110kDa protein 1	
Hsp90aa1	0	0	0	1	0	heat shock protein 90, alpha, class A member 1	
Hsp90b1	0	0	1	0	0	heat shock protein 90, beta, member 1	
Hspa1a	1	0	0	0	0	heat shock protein 1A	
Hspa1b	1	0	2	0	0	heat shock protein 1B; Hsp70	18
Hspa5	0	0	2	0	0	heat shock protein 5	
Hspb1	0	0	0	0	2	heat shock protein 1	18
Hspb6	0	0	0	0	1	heat shock protein, B6	
Htr4	0	0	1	0	0	5 hydroxytryptamine receptor 4	
Ibrdc1	1	0	0	0	0	ring finger protein 217; Rnf217	
Ibrdc3	0	0	1	0	0	ring finger protein 19B; Rnf19b	
Icam1	0	0	0	0	1	intercellular adhesion molecule 1	
Id1	1	0	0	0	0	inhibitor of DNA binding 1	
Id2	1	0	1	0	0	inhibitor of DNA binding 2	44
Idi1	2	0	0	0	0	isopentenyl-diphosphate isomerase1	
Ids	0	0	0	0	1	iduronate 2-sulfatase	
Ier2	1	0	0	0	0	immediate early response 2; ch1, pip92	
Ier3	0	0	0	1	0	immediate early response 3	
Ier5	2	0	0	0	0	immediate early response 5	
Ifi202b	0	0	1	0	0	interferon activated gene 202B	
Ifit1	0	0	0	0	1	interferon-induced protein with tetratricopeptide repeats 1	
Ifit2	0	0	0	0	1	interferon-induced protein with tetratricopeptide repeats 2	
Ifit3	0	0	0	0	1	interferon-induced protein with tetratricopeptide repeats 3	
Ifitm3	0	0	0	0	1	interferon induced transmembrane protein 3	
Ifrd1	1	1	0	0	0	interferon-related developmental regulator1	
Igtp	0	0	0	0	1	interferon gamma induced GTPase	
Iigp1	0	0	0	0	2	interferon inducible GTPase 1	
Il13ra1	0	0	0	0	4	interleukin 13 receptor, alpha 1	
Il33	0	0	0	0	1	interleukin 33	
Ilf2	0	0	0	1	0	interleukin enhancer binding factor 2	
Impact	0	1	0	0	0	imprinted and ancient	
Inhba	1	0	0	0	0	inhibin, beta A; EDF, FRP	
Ints8	0	0	0	0	1	integrator complex subunit 8	
Iqgap1	0	0	0	0	2	IQ motif containing GTPase activating protein 1	
Irf2bp2	0	1	0	0	0	interferon regulatory factor 2 binding protein 2	
Irgm	0	0	0	0	1	immunity-related GTPase family M member 1	
Irs2	1	0	0	0	0	insulin receptor substrate 2	
Isgf3g	0	0	0	0	1	interferon regulatory factor 9	
Itgav	0	0	1	1	0	integrin, alpha V; vitronectin receptor	45
Itgb2	0	0	0	0	1	integrin beta 2; LCAMB, Lfa1, MF17	
Itpkc	0	0	1	0	0	inositol 1,4,5-trisphosphate 3-kinase C	
Ivns1abp	1	0	0	0	0	influenza virus NS1A binding protein	
Jak1	0	0	0	1	0	Janus kinase 1	
Jarid2	3	1	0	0	0	jumonji, AT rich interactive domain 2	
Jmjd3	2	1	1	0	0	jumonji domain containing 3	
Jph1	0	2	0	0	0	junctophilin 1	
Jun	2	0	0	0	0	AP1, c-Jun	46
Junb	0	1	0	0	0	Jun-B oncogene	31
Kbtbd8	0	0	0	0	1	kelch repeat and BTB domain containing 8	

Kcna4	1	0	1	0	0	potassium voltage-gated channel, shaker-related subfamily, member 4	
Kcnd3	0	0	0	1	0	potassium voltage-gated channel, Shal-related family, member 3	
Kcnf1	0	1	0	0	0	potassium voltage-gated channel, subfamily F, member 1	
Kcnip3	0	0	1	1	0	Kv channel interacting protein 3	
Kcnj4	1	0	0	0	0	potassium inwardly-rectifying channel, subfamily J, member 4	
Kcnk1	1	0	1	0	0	potassium channel, subfam. K, member1	
Kcnn2	1	0	0	0	0	potassium intermediate/small conductance calcium-activ. channel, subfam. N, member 2	
Kcnq2	0	0	0	0	1	potassium voltage-gated channel, subfamily Q, member 2	
Kif18a	0	0	0	1	0	kinesin family member 18A	
Kif1b	0	1	0	0	0	kinesin family member 1B	
Kif5c	0	0	0	1	0	kinesin family member 5C	
Kitl	0	1	0	0	0	kit ligand	
Klf10	0	0	1	0	0	Kruppel-like factor 10	
Klf2	0	1	0	0	0	Kruppel-like factor 2	
Klf4	1	1	0	0	0	Kruppel-like factor 4	
Klf6	0	0	0	5	0	Kruppel-like factor 6	
Klhl24	0	0	0	0	1	kelch-like 24; kainate receptor interacting protein for GluR6	
Klk6	0	0	0	0	1	kallikrein related-peptidase 6; neurosin	
Kns2	0	0	0	1	0	kinesin light chain 1	
Kpna1	0	0	0	1	0	karyopherin alpha 1; m-importin-alpha-S1	
Kpnb1	0	0	0	1	0	karyopherin (importin) beta 1	
Kras	0	0	0	1	0	v-Ki-ras2 Kirsten rat sarcoma viral oncogene homolog	
Krt2	0	0	1	0	0	keratin gene complex 2, basic	
Krt75	0	0	1	0	0	keratin 75; cytokeratin KRT2-6HF	
L3mbtl3	0	0	1	0	0	l(3)mbt-like 3; MBT-1	
LOC546201	1	0	0	0	0	PRAME family member 8	
LOC554327	0	0	2	0	0	RIKEN cDNA 2610042L04 gene	
LOC620695	0	1	0	0	0	predicted gene, OTTMUSG00000014994	
LOC634748	0	0	0	0	1	similar to transmembrane trafficking protein	
LOC666231	0	0	0	1	0	predicted gene, EG666231	
LOC671237	0	0	0	1	0	similar to Putative RNA-binding protein 3	
Lactb2	0	0	0	0	1	lactamase, beta 2	
Lamp2	0	0	0	1	0	lysosomal-associated membr. protein 2	
Lancl2	0	0	0	1	0	LanC -like 2	
Laptm5	0	0	0	0	1	lysosomal-associated protein transmembrane 5	
Lats2	1	0	0	0	0	large tumor suppressor 2	
Lbh	2	0	0	0	0	Lbh, limb-bud and heart	
Lcn2	1	0	0	0	0	lipocalin 2; NGAL	
Lcp1	0	0	0	0	2	lymphocyte cytosolic protein 1	
Lcp2	0	0	0	0	1	lymphocyte cytosolic protein 2	
Lgals1	0	0	0	0	2	lectin, galactose binding, soluble 1	
Lgals3	0	0	1	0	0	lectin, galactose binding, soluble 3	47
Lgals7	0	0	2	0	0	lectin, galactose binding, soluble 7	
Lgmn	0	0	0	0	1	legumain	
Lhfp	0	0	1	0	0	lipoma HMGIC fusion partner	
Lif	0	0	1	0	0	leukemia inhibitory factor	47
Lilrb4	0	0	1	0	0	leukocyte immunoglobulin-like receptor, subfamily B, member 4	
Lin7c	0	0	0	0	1	lin-7 homolog C	
Lingo1	0	0	2	0	0	leucine rich repeat and Ig domain containing1	48
Litaf	0	0	0	1	0	LPS-induced TN factor	
Lix1l	0	0	1	0	0	Lix1-like	
Lman2	0	0	1	0	0	lectin, mannose-binding 2	
Lmo2	0	0	1	0	0	LIM domain only 2	49

Lmo7	1	0	0	0	0	LIM domain only 7	
Loh11cr2a	0	0	0	0	1	loss of heterozygosity, 11, chromosomal region 2, gene A homolog	
Lonrf3	2	0	0	0	0	LON peptidase N-terminal domain and ring finger 3	
Loxl1	0	0	0	0	1	lysyl oxidase-like 1	
Lphn3	1	0	10	0	0	latrophilin 3	
Lpin2	0	0	1	0	0	lipin 2	
Lrg1	0	0	0	1	0	leucine-rich alpha-2-glycoprotein 1	
Lrig1	0	0	2	0	0	leucine-rich repeats and immunoglobulin-like domains 1	
Lrp1b	0	0	0	1	0	low density lipoprotein-related protein 1B	
Lrpap1	0	0	1	0	0	low density lipoprotein receptor-related protein associated protein 1; RAP	
Lrrc48	0	0	2	0	0	leucine rich repeat containing 48	
Lrrfip1	1	0	0	0	0	leucine rich repeat interacting protein 1	
Lrrtm3	0	0	0	0	1	leucine rich repeat transmembrane neuronal3	
Lrtm2	0	0	1	0	0	leucine-rich repeats and transmembrane domains 2	
Lta4h	0	0	0	1	0	leukotriene A4 hydrolase	
Ly6a	0	0	1	0	0	lymphocyte antigen 6 complex, locus A	
Ly6g6e	0	0	0	0	1	lymphocyte antigen 6 complex, locus G6E	
Ly86	0	0	0	0	1	lymphocyte antigen 86	
Maff	1	0	0	0	0	v-maf musculoaponeurotic fibrosarcoma oncogene family, protein F	
Mafk	0	1	0	0	0	v-maf musculoaponeurotic fibrosarcoma oncogene family, protein K	
Magoh	1	0	0	0	0	mago-nashi homolog	
Malat1	0	2	0	0	0	metastasis associated lung adenocarcinoma transcript 1	
Map1lc3a	0	1	0	0	0	microtubule-associated protein 1 light chain 3 alpha	
Map3k5	1	0	0	0	0	mitogen-activated protein kinase kinase kinase 5	50
Map3k6	1	0	0	0	0	mitogen-activated protein kinase kinase kinase 6	
Mapk12	0	0	0	0	1	mitogen-activated protein kinase 12	
Mapk4	0	0	1	0	0	mitogen-activated protein kinase 4	
Mapk6	0	0	1	0	0	mitogen-activated protein kinase 6	
Mapkapk2	1	0	0	0	0	MAP kinase-activated protein kinase 2	51
Mapkapk3	0	0	2	0	0	mitogen-activated protein kinase-activated protein kinase 3	
March6	0	1	0	0	0	membrane-associated ring finger 6	
Mast4	0	1	0	0	0	microtubule associated serine/threonine kinase family member 4	
Mat2a	0	1	0	0	0	methionine adenosyltransferase II, alpha	
Matr3	0	0	0	1	0	matrin 3	
Mcf2	0	0	0	0	1	mcf.2 transforming sequence	
Mcl1	1	0	4	1	0	myeloid cell leukemia sequence 1	
Mcm2	0	0	0	0	1	minichromosome maintenance deficient 2	
Mcm3	0	0	0	0	1	minichromosome maintenance deficient 3	
Mcm5	0	0	0	0	1	minichromosome maintenance deficient 5	
Megf11	0	0	1	0	0	multiple EGF-like-domains 11	
Mest	0	1	0	0	0	mesoderm specific transcript; Peg1	
Mfap3l	0	0	0	0	1	microfibrillar-associated protein 3-like	
Mfsd2	1	0	0	0	0	major facilitator superfamily domain containing 2	
Mgst3	0	1	0	0	0	microsomal glutathione S-transferase 3	
Mid1	0	0	1	0	0	midline 1; TRIM18	
Midn	1	0	0	0	0	midnolin	
Mina	0	1	0	0	0	myc induced nuclear antigen	
Minpp1	0	0	0	1	0	multiple inositol polyphosphate histidine phosphatase 1	
Mknk2	0	1	0	0	0	MAP kinase-interacting serine/threonine kinase 2	
Mlf1	0	1	0	0	0	myeloid leukemia factor 1	
Mmd	1	0	0	0	0	monocyte to macrophage differentiation-associated	
Mobp	0	1	0	0	0	myelin-associated oligodendrocytic basic protein	
Mpp6	0	0	1	0	0	membrane protein, palmitoylated 6	

Mrps6	0	0	0	0	2	mitochondrial ribosomal protein S6	
Ms4a6d	0	0	0	0	2	membrane-spanning 4-domains, subfamily A, member 6D	
Msn	0	0	0	0	2	moesin	
Msr2	0	0	0	0	1	macrophage scavenger receptor 2	
Mt1	2	0	0	0	0	metallothionein 1	52
Mt2	1	0	0	0	0	metallothionein 2	52
Mtap2	0	0	0	2	0	microtubule-associated protein 2; MAP2	53,54
Mtch2	0	0	0	0	1	mitochondrial carrier homolog 2;Hspc032	
Mtmr11	0	0	0	0	1	myotubularin related protein 11	
Mtpn	0	0	0	1	0	myotrophin	
Mvp	0	0	0	0	1	major vault protein; VAULT1	
Myc	0	0	0	1	0	myelocytomatosis oncogene	
Myd88	0	0	0	0	1	myeloid differentiation primary response gene 88	
Myef2	0	0	0	1	0	myelin basic protein expression factor 2	
Myh9	1	0	0	0	0	myosin, heavy polypeptide 9	
Mynn	0	0	0	1	1	myoneurin	
Narf	0	0	0	1	0	nuclear prelamin A recognition factor	55
Ncan	0	0	0	1	0	neurocan	56
Ndnl2	0	1	0	0	0	necdin-like 2	
Ndph	0	0	0	0	1	Norrie disease homolog; norrin	
Nebl	0	0	0	0	1	nebulette; actin-binding Z-disc protein	
Nedd4	0	0	0	0	1	neural precursor cell expressed, developmentally down-regulated gene 4	
Nedd9	1	0	2	0	0	neural precursor cell expressed, developmentally down-regulated 9	
Nefh	0	0	1	0	0	neurofilament, heavy polypeptide; NF-H	
Nefl	0	1	0	0	0	neurofilament, light polypeptide	
Nek6	0	0	0	0	2	NIMA-related expressed kinase 6	
Nfil3	1	0	0	0	0	nuclear factor, interleukin 3 regulated	
Nfkbiz	1	1	0	0	0	nuclear factor of kappa light polypeptide gene enhancer in B-cells inhibitor	
Nipa2	0	0	0	1	0	non imprinted in Prader-Willi/Angelman syndrome 2 homolog	
Nipsnap3a	0	0	0	0	1	nipsnap homolog 3A	
Nln	0	0	0	0	1	neurolysin; neurotensin endopeptidase	
Nmnat2	0	1	0	0	0	nicotinamide nucleotide adenylyltransferase2	
Noc4l	0	0	0	0	1	nucleolar complex associated 4 homolog	
Nol4	0	1	0	0	0	nucleolar protein 4	
Nolc1	1	0	0	0	0	nucleolar and coiled-body phosphoprotein 1	
Npas4	0	1	0	0	0	HLH-PAS transcription factor NXF	29,57
Nptx1	0	0	2	0	0	neuronal pentraxin 1	
Nptx2	2	0	0	0	0	neuronal pentraxin II, NARP	58,59
Nptxr	0	0	1	0	0	neuronal pentraxin receptor	
Npy	0	0	1	0	0	neuropeptide Y	60
Nr1d2	0	0	0	0	1	nuclear receptor subfamily 1, gr D, member 2	
Nr4a1	0	1	0	0	0	nuclear receptor subfamily 4, gr. A, member 1(=NGFI-B, nur77)	61
Nr4a2	1	3	0	0	0	nuclear receptor subfamily 4, group A, member 2; nurr1	62
Nr4a3	0	1	0	0	0	Nor1	63
Nrn1	1	0	0	0	0	neuritin 1 (cpg15)	64
Nrp1	1	1	0	0	0	neuropilin 1	
Ntrk2	0	1	0	0	0	neurotrophic tyrosine kinase, receptor, type 2 (trkB)	65
Nts	1	0	0	0	0	neurotensin	
Nudcd2	0	0	0	0	1	NudC domain containing 2	
Nudt6	0	0	1	0	0	nudix-type motif 6	
Nudt9	0	0	1	0	0	nudix-type motif 9	
Nupr1	0	0	0	0	2	nuclear protein 1	
Oasl2	0	0	0	0	1	2'-5' oligoadenylate synthetase-like 2	

Odc1	0	0	0	2	0	ornithine decarboxylase 1	
Olfm4	0	0	1	0	0	olfactomedin 4	
Olfml3	0	0	0	0	1	olfactomedin-like 3	
Onecut2	0	1	0	0	0	one cut domain, family member 2	
Opa1	0	0	0	2	0	optic atrophy 1 homolog	
Osbpl6	2	0	0	0	0	oxysterol binding protein-like 6	
Osgin2	1	0	1	0	0	oxidative stress induced growth inhibitor family member 2	
Osmr	0	0	0	2	0	oncostatin M receptor	
Otub1	0	2	0	0	0	OTU domain, ubiquitin aldehyde binding 1	
P2ry6	0	0	0	0	1	pyrimidinergic receptor P2Y, G-protein coupled, 6	
P4ha1	0	0	0	1	0	procollagen-proline	
Pabpc4	1	0	0	1	0	poly(A) binding protein, cytoplasmic 4	
Pabpn1	0	1	0	0	0	poly(A) binding protein, nuclear 1	
Pafah1b2	0	0	0	1	0	platelet-activating factor acetylhydrolase, isoform 1b, alpha2 subunit	
Pak6	0	0	0	2	0	p21(CDKN1A)-activated kinase 6	
Pak7	0	0	0	1	0	p21 (CDKN1A)-activated kinase 7	
Pam	0	1	0	0	0	peptidylglycine alpha-amidating monooxygenase	17
Pappa	0	0	1	0	0	pregnancy-associated plasma protein A	
Paqr9	0	0	1	0	0	progestin and adipoQ receptor family member IX	
Parp3	0	0	0	0	2	poly (ADP-ribose) polymerase family, member 3	
Pcdh17	0	1	0	0	0	protocadherin 17	
Pcdh8	2	0	0	0	0	protocadherin 8, ARCADLIN	66
Pcdh9	2	0	0	0	0	protocadherin 9	
Pcmt1	0	0	1	0	0	protein-L-isoaspartate (D-aspartate) O-methyltransferase 1; PIMT	
Pcnx	2	0	0	1	0	pecanex homolog	
Pcsk1	1	0	0	0	0	proprotein convertase subtilisin/kexin type 1= NEC1, PC1, PC3, SPC3	36
Pctk3	0	0	0	0	1	PCTAIRE-motif protein kinase 3	
Pde4d	0	1	0	0	0	phosphodiesterase 4D, cAMP specific	
Pde4dip	0	1	2	0	0	phosphodiesterase 4D interacting protein	
Pdia3	0	0	0	1	0	protein disulfide isomerase associated 3	
Pdia6	0	0	1	0	0	protein disulfide isomerase associated 6	
Pdlim1	0	0	1	0	0	PDZ and LIM domain 1 (elfin); (Clim1)	
Pdlim4	0	0	0	0	1	PDZ and LIM domain 4	
Pdlim5	1	0	0	3	0	PDZ and LIM domain 5	
Pdyn	1	0	0	0	0	prodynorphin; beta-neoendorphin-dynorphin; proenkephalin B	67,68
Pdzd2	0	0	1	0	0	PDZ domain containing 2; Papin,	
Peli1	0	3	0	0	0	pellino 1	
Penk1	1	0	0	0	0	preproenkephalin 1	67,68
Per1	0	1	0	0	0	stress and light induced/period homolog1	
Pex5	1	0	0	1	0	peroxisomal biogenesis factor 5	
Pfkfb2	0	0	0	1	0	6-phosphofructo-2-kinase/fructose-2,6-biphosphatase 2	
Pfkfb3	1	0	0	1	0	6-phosphofructo-2-kinase/fructose-2,6-biphosphatase 3	
Pfn1	0	0	0	1	0	profilin 1	
Pglyrp1	1	0	0	0	0	peptidoglycan recognition protein 1	
Phactr3	0	1	0	0	0	phosphatase and actin regulator 3	
Phex	0	0	1	0	0	phosphate regulating gene with homologies to endopeptidases on the X chromosome	
Phf16	0	0	1	0	0	PHD finger protein 16	
Phf6	0	1	0	0	0	PHD finger protein 6	
Pik3r1	0	1	0	0	0	phosphatidylinositol 3-kinase, regulatory subunit, polypeptide 1	
Pik4ca	0	0	1	0	0	phosphatidylinositol 4-kinase, catalytic, alpha polypeptide	
Pim1	2	0	0	0	0	proviral integration site 1; non-specific serine/threonine protein kinase	69,70
Pim3	0	1	0	0	0	proviral integration site 3; Kid1	69
Pkia	0	0	0	0	1	protein kinase inhibitor, alpha	

Plagl1	1	0	0	0	0	leiomorphic adenoma gene-like 1; Zac1	71
Plat	0	0	0	1	0	plasminogen activator, tissue; tPA	72
Plaur	0	0	0	1	0	plasminogen activator, urokinase receptor, UPAR, URKR	73
Plce1	0	0	1	0	0	phospholipase C, epsilon 1	
Plek	0	0	0	0	3	pleckstrin	
Plekha2	0	0	2	0	0	pleckstrin homology domain-containing, family A member 2	
Plekhh2	0	1	0	0	0	pleckstrin homology domain containing, family H member 2	
Plk2	1	0	0	0	0	polo-like kinase 2; Snk	74
Plk3	1	0	0	0	0	polo-like kinase 3; Fnk	74
Pln	0	1	0	0	0	phospholamban	
Plscr2	0	0	0	0	1	phospholipid scramblase 2	
Pmaip1	1	0	0	0	0	phorbol-12-myristate-13-acetate-induced protein 1	
Pmvk	1	0	0	0	0	phosphomevalonate kinase	
Pnoc	0	0	1	0	0	prepronociceptin; orphanin FQ	
Podxl2	0	0	0	1	0	podocalyxin-like 2	
Polr3e	0	1	0	0	0	polymerase (RNA) III polypeptide E	
Pp11r	0	0	0	0	1	placental protein 11 related	
Ppie	0	0	0	0	1	peptidylprolyl isomerase E (cyclophilin E)	
Ppm1b	0	0	0	1	0	protein phosphatase 1B	
Ppm1h	0	1	1	0	0	protein phosphatase 1H	
Ppp1r12a	0	0	0	1	0	protein phosphatase 1, regulatory subunit 12A myosin phosphatase	
Ppp1r16b	0	1	0	0	0	protein phosphatase 1, regulatory (inhibitor) subunit 16B	
Ppp1r3c	0	0	1	1	0	protein phosphatase 1, regulatory (inhibitor) subunit 3C	
Ppp1r3g	0	1	0	0	0	protein phosphatase 1, regulatory (inhibitor) subunit 3G	
Ppp2r1b	0	1	0	0	0	protein phosphatase 2, regulatory subunit A, beta isoform	
Ppp2r2b	0	0	0	1	0	protein kinase, cAMP dependent regulatory, type II alpha	
Ppp2r5c	0	0	1	0	0	protein phosphatase 2, regulatory subunit B (B56), gamma isoform	
Ppp3cb	0	0	0	1	0	protein phosphatase 3, catalytic subunit, beta isoform	
Ppp3r1	0	0	0	1	0	protein phosphatase 3, regulatory subunit B, alpha isoform; Cnb1	
Ppp4r2	0	0	2	0	0	protein phosphatase 4, regulatory subunit 2	
Prkaa1	0	0	0	0	1	protein kinase, AMP-activated, alpha 1 catalytic subunit	
Prkacb	0	0	0	1	0	protein kinase, cAMP dependent, catalytic, beta	
Prkar1a	0	0	0	1	0	protein kinase, cAMP dependent regulatory, type I, alpha	
Prkar2a	0	1	0	0	0	protein kinase, cAMP dependent regulatory, type II alpha	
Prlr	0	0	2	0	1	prolactin receptor	
Prokr2	0	0	2	0	0	prokineticin receptor 2; EG-VEGRF2	
Pros1	0	0	0	0	1	protein S (alpha)	
Prosc	1	0	0	0	0	proline synthetase co-transcribed homolog	
Prpf39	0	0	0	1	0	PRP39 pre-mRNA processing factor 39 homolog	
Prss23	0	0	0	2	1	protease, serine, 23	
Psmb2	0	0	0	1	0	proteasome (prosome, macropain) subunit, beta type 2	
Psmc6	0	0	0	0	1	proteasome (prosome, macropain) 26S subunit, ATPase, 6	
Psmd11	0	0	0	1	0	proteasome (prosome, macropain) 26S subunit, non-ATPase, 11	
Psmd14	0	1	0	0	0	proteasome (prosome, macropain) 26S subunit, non-ATPase, 14	
Psmd2	0	0	0	1	0	proteasome (prosome, macropain) 26S subunit, non-ATPase, 2	
Ptbp1	0	0	0	1	0	polypyrimidine tract binding protein 1	
Ptger2	0	0	1	0	0	prostaglandin E receptor 2 (subtype EP2)	
Ptger4	0	0	0	1	0	prostaglandin E receptor 4 (subtype EP4)	
Ptgs2	2	0	0	0	0	prostaglandin-endoperoxide synthase 2; cyclooxygenase 2 COX2	75
Pthlh	2	0	0	0	0	parathyroid hormone-like peptide	
Ptk2	0	0	1	0	0	focal adhesion kinase	
Ptma	0	0	0	1	0	prothymosin, alpha	
Ptp4a2	0	0	1	0	0	protein tyrosine phosphatase 4a2	

Ptpn12	2	0	0	1	0	protein tyrosine phosphatase, non-receptor type 12	
Ptprc	0	0	0	0	1	protein tyrosine phosphatase, receptor type, C	
Ptprj	0	0	0	1	0	protein tyrosine phosphatase, receptor type, J	
Ptprk	1	0	0	0	0	protein tyrosine phosphatase, receptor type, K	
Ptprs	0	1	0	0	0	protein tyrosine phosphatase, receptor type, S	
Ptrf	1	0	0	0	0	polymerase I and transcript release factor	
Ptx3	0	0	0	1	0	pentraxin-related gene	76
Pvr	1	0	0	0	0	poliovirus receptor nectin-like-5	
Pxdn	0	0	1	0	0	peroxidasin homolog	
Pycard	0	0	0	0	1	PYD and CARD domain containing	
R3hdm1	2	4	0	0	0	R3H domain containing	
Rab10	0	0	0	0	1	RAB10, member RAS oncogene family	
Rab1b	0	0	0	1	0	RAB1B, member RAS oncogene family	
Rab27a	0	0	0	0	1	RAB27A, member RAS oncogene family	
Rabgap1	0	0	0	1	0	RAB GTPase activating protein 1	
Rabgef1	0	0	0	1	0	RAB guanine nucleotide exchange factor (GEF) 1	
Rai2	0	0	1	0	0	retinoic acid induced 2	
Ranbp1	0	0	0	1	0	RAN binding protein 1	
Ranbp2	2	0	0	0	0	RAN binding protein 2, nucleoporin 35	
Ranbp5	0	0	0	1	0	importin 5; Ipo5	
Rap1b	0	0	0	1	0	RAS related protein 1b	
Rasd1	0	1	0	0	0	RAS, dexamethasone-induced 1	
Rasgef1b	0	1	0	0	0	RasGEF domain family, member 1B;	
Rasgrf1	1	0	0	0	0	RAS protein-specific guanine nucleotide-releasing factor 1	
Rasl10a	0	0	1	0	0	RAS-like, family 10, member A	
Rasl11a	0	1	0	0	0	RAS-like, family 11, member A	
Rassf5	0	0	1	0	0	Ras association domain family member 5	
Rbbp7	2	0	0	0	0	retinoblastoma binding protein 7	
Rbm4b	0	1	0	0	0	RNA binding motif protein 4B	
Rbpsuh	0	0	2	1	0	recombination signal binding protein for immunoglobulin kappa J region	
Rcc2	0	0	0	1	0	regulator of chromosome condensation 2	
Refbp2	0	0	0	1	0	RNA and export factor binding protein 2	
Rem2	2	0	0	0	0	rad and gem related GTP binding protein 2	
Reps2	0	0	0	0	1	RALBP1 associated Eps domain containing protein 2	
Rest	0	0	1	0	0	RE1-silencing transcription factor; NRSF	77
Rfc4	0	0	0	0	1	replication factor C (activator 1) 4	
Rfwd2	1	0	1	0	0	ring finger and WD repeat domain 2	
Rfx4	1	0	0	0	0	winged-helix transcription factor	
Rgs2	3	0	0	0	0	regulator of G-protein signaling 2	78
Rgs20	0	0	0	0	1	regulator of G-protein signaling 20	
Rgs4	3	0	0	0	0	regulator of G-protein signaling 4	79,80
Rgs7	0	0	1	0	0	regulator of G protein signaling 7	
Rgs7bp	0	0	0	1	0	regulator of G-protein signalling 7 binding protein	
Rgs8	0	0	1	0	0	regulator of G-protein signaling 8	
Rhbdf1	0	0	1	0	0	rhomboid family 1	
Rheb	1	0	0	0	0	Ras homolog enriched in brain	81
Rhob	0	0	0	1	0	ras homolog gene family, member B	
Rhoc	0	0	0	1	1	ras homolog gene family, member C	
Rhoj	0	0	0	1	0	ras homolog gene family, member J	
Rhoq	0	0	1	0	0	ras homolog gene family, member Q	
Rims4	0	0	1	0	0	regulating synaptic membrane exocytosis 4	
Rkhd2	0	0	1	0	0	mex3 homolog C	
Rkhd3	0	0	0	0	1	mex3 homolog B	

Rnd3	2	0	0	0	0	similar to Rho-related GTP-binding protein RhoE (Rho family GTPase 3)	
Rnf12	1	0	0	0	0	RLIM, ring finger protein 12	
Rnf125	0	0	0	1	0	ring finger protein 125	
Rnf128	0	1	0	0	0	ring finger protein 128; GRAIL	
Rnpc3	0	0	0	1	0	RNA-binding region containing 3	
Rp2h	0	0	0	0	2	retinitis pigmentosa 2 homolog	
Rprm	0	0	0	0	1	reprimo	
Rprml	1	0	0	0	0	reprimo-like	
Rps6ka3	1	0	0	0	0	ribosomal protein S6 kinase polypeptide3	
Rps6ka6	0	0	1	0	0	ribosomal protein S6 kinase polypeptide6	
Rrad	1	0	0	0	0	Ras-related associated with diabetes	
Rras2	0	2	0	0	0	related RAS viral oncogene homolog 2	
Rrm2b	0	0	0	0	1	ribonucleotide reductase M2 B	
Rtn4rl1	0	0	1	0	0	reticulon 4 receptor-like 1; nogo receptor-like 2; nogo-66 receptor homolog 2	
Rtn4rl2	0	0	1	0	0	reticulon 4 receptor-like 2; nogo receptor-like 3; nogo-66 receptor homolog 1	
Rtp4	0	0	0	0	1	receptor transporter protein 4	
Rwdd3	0	0	2	0	0	RWD domain containing 3	
S100a10	0	0	0	0	2	S100 calcium binding protein A10	
S100a11	0	0	0	0	1	S100 calcium binding protein A11	
S100a16	0	0	0	0	2	S100 calcium binding protein A16	
S100a4	0	0	0	0	1	S100 calcium binding protein A4	
S100a6	0	0	0	0	1	S100 calcium binding protein A6	
S3-12	1	0	0	0	0	plasma membrane associated protein	
Samsn1	0	0	0	0	1	SAM domain, SH3 domain and nuclear localization signals, 1	
Sap18	1	0	0	0	0	Sin3-associated polypeptide 18	
Sap30	1	0	0	0	0	sin3 associated polypeptide	
Sat1	1	0	0	0	0	spermidine/spermine N1-acetyl transferase 1	
Sbno1	0	0	0	1	0	strawberry notch homolog 1	
Sbno2	0	0	0	2	0	strawberry notch homolog 2	
Sc4mol	0	0	0	1	0	sterol-C4-methyl oxidase-like	
Sc5d	1	0	0	0	0	sterol-C5-desaturase	
Scamp1	0	0	0	1	0	secretory carrier membrane protein 1	
Scd1	0	0	0	1	0	stearoyl-Coenzyme A desaturase 1	
Scg2	1	0	0	0	0	secretogranin II; secretoneurin	18,82
Scgb3a1	0	0	0	0	1	secretoglobin, family 3A, member 1	
Schip1	0	1	0	1	0	schwannomin interacting protein 1	
Sdc1	0	0	0	0	2	syndecan 1	18
Sdc4	0	1	0	0	0	syndecan 4	
Sdcbp	0	0	0	1	0	syndecan binding protein; syntenin	
Sdcbp2	0	0	1	0	0	syndecan binding protein (syntenin) 2	
Sdf2l1	0	0	1	0	0	stromal cell-derived factor 2-like 1	
Sec61a1	0	0	0	2	0	Sec61 alpha 1 subunit	
Seh1l	0	0	0	0	1	SEH1-like	
Sema3e	0	0	2	1	0	semaphorin 3E	
Senp2	0	0	0	3	0	SUMO/sentrin specific peptidase 2	
Sep 8	0	0	0	1	0	Septin 8, GTP-binding	
Sepw1	0	1	0	0	0	selenoprotein W, muscle 1	
Serinc1	0	0	0	1	1	serine incorporator 1	
Serinc2	0	0	1	0	0	serine incorporator 2	
Serpina3n	0	0	0	0	1	serine peptidase inhibitor, cl. A, member 3N	
Serpine1	0	0	0	1	0	serine peptidase inhibitor, cl. E, member 1; Plasminogen activator inhibitor-1 precursor (PAI-1)	83
Serpinf2	0	0	0	0	1	serine (or cysteine) peptidase inhibitor, cl. F, member 2; alpha-2-antiplasmin	
Serpinh1	0	0	0	1	0	serine (or cysteine) peptidase inhibitor, cl. H, member 1	

Sertad1	1	0	0	0	0	SERTA domain containing 1	
Setd8	0	0	2	0	0	SET domain containing 8	
Sez6l	1	0	0	0	0	seizure related 6 homolog like	84
Sf3b1	1	0	0	0	0	splicing factor 3b, subunit 1	
Sfpq	0	0	1	1	0	splicing factor proline/glutamine rich	
Sfrs1	0	0	0	1	1	splicing factor, arginine/serine-rich 1	
Sfrs3	0	0	1	0	0	splicing factor, arginine/serine-rich 3	
Sgk	1	0	0	0	0	serum/glucocorticoid regulated kinase 1	
Sgk3	1	0	0	0	0	serum/glucocorticoid regulated kinase 3	
Sgpl1	0	0	0	1	0	sphingosine phosphate lyase 1	
Sh2d5	0	0	1	0	0	SH2 domain containing 5	
Sh3pxd2b	0	0	1	0	0	SH3 and PX domains 2B	
Shc2	0	0	0	1	0	src homology 2 domain-containing transforming protein C2	
Siah1b	0	0	0	0	1	seven in absentia 1B	
Siah2	0	2	0	0	0	seven in absentia 2	
Sin3b	0	1	0	0	0	transcriptional regulator, SIN3B	
Ski	0	0	1	0	0	ski sarcoma viral oncogene homolog	
Skil	0	0	1	0	0	SKI-like	
Slc10a4	0	0	0	0	1	solute carrier family 10, member 4	
Slc10a7	0	0	1	0	0	solute carrier family 10, member 7	
Slc14a1	0	0	0	0	2	solute carrier family 14, member 1	
Slc16a1	0	0	0	1	0	solute carrier family 16, member 1	
Slc17a7	0	1	0	0	0	solute carrier family 17, member 7; Vglut1	
Slc1a3	0	0	0	1	0	solute carrier family 1 member 3; Eaat1	
Slc20a1	0	0	1	0	0	solute carrier family 20, member 1	
Slc22a4	0	0	0	1	0	solute carrier family 22, member 4	
Slc23a2	0	0	0	2	0	solute carrier family 23, member 2	
Slc24a4	0	0	1	0	0	solute carrier family 24, member 4; NCKX4	
Slc25a25	0	1	0	0	0	solute carrier family 25, member 25	
Slc25a3	0	1	0	0	0	solute carrier family 25, member 3	
Slc25a36	0	0	0	1	0	solute carrier family 25, member 36	
Slc2a1	0	3	0	0	0	solute carrier family 2, member 1; Glut1	
Slc2a3	0	0	0	2	0	solute carrier family 2, member 3; Glut3	
Slc35f3	0	0	1	0	0	solute carrier family 35, member F3	
Slc36a1	0	0	1	0	0	solute carrier family 36, member 1	
Slc39a10	0	0	0	0	2	solute carrier family 39, member 10	
Slc3a2	1	0	0	0	0	solute carrier family 3, member 2	
Slc41a2	0	0	1	0	0	solute carrier family 41, member 2	
Slc44a3	0	0	0	0	1	solute carrier family 44, member 3	
Slc6a11	0	1	0	0	0	solute carrier family 6, member 11	
Slc6a17	1	0	0	0	0	solute carrier family 6, member 17	
Slc6a6	0	1	0	0	0	solute carrier family 6, member 6	
Slc6a8	1	0	1	0	0	solute carrier family 6, member 8	
Slc7a14	0	1	0	0	0	solute carrier family 7, member 14	
Slc7a5	1	0	0	0	0	solute carrier family 7, member 5	
Smad7	1	2	0	0	0	MAD homolog 7	
Smap1l	1	0	0	0	0	stromal membrane-associated GTPase-activating protein 2	
Smarca5	1	0	0	0	0	SWI/SNF related, matrix associated, actin dependent regulator of chromatin, subfamily a, member 5	
Smek1	0	0	0	1	0	SMEK homolog 1, suppressor of mek1	
Smoc2	0	0	0	0	1	SPARC related modular calcium binding2	
Smyd4	1	0	0	0	0	SET and MYND domain containing 4	
Snf1lk	0	2	0	0	0	SNF1-like kinase; salt-inducible protein kinase	
Snf1lk2	0	1	0	0	0	salt induceable kinase 2	

Snx13	0	0	0	1	0	sorting nexin 13	
Socs3	0	0	0	3	0	suppressor of cytokine signaling 3	85
Son	0	0	0	1	0	Son DNA binding protein	
Sorcs3	0	0	2	0	0	sortilin-related VPS10 domain containing receptor 3	86
Sox11	0	0	6	0	0	SRY-box containing gene 11	87
Sox2	0	0	0	0	1	SRY-box containing gene 2	
Sp3	0	0	0	0	1	trans-acting transcription factor 3	
Spata5l1	0	0	0	0	1	spermatogenesis associated 5-like 1	
Sphk1	0	0	1	0	0	sphingosine kinase 1	
Spock3	0	0	0	0	1	sparc/osteonectin, cwcv and kazal-like domains proteoglycan 3	
Spop	0	0	0	1	0	speckle-type POZ protein	
Spp1	0	0	1	0	0	secreted phosphoprotein 1; osteopontin	88
Spred1	0	0	0	1	1	sprouty-related, EVH1 domain cont. 1	
Spred2	2	0	1	0	0	sprouty-related, EVH1 domain cont. 2	
Spry1	0	0	0	0	1	sprouty homolog 1	
Spsb1	1	1	0	0	0	SPRY domain-containing SOCS box protein SSB-1	
Spty2d1	1	0	0	0	0	SPT2, Suppressor of Ty, domain containing 1	
Sqle	1	0	0	0	0	squalene epoxidase	
Srebf2	0	1	0	0	0	sterol regulatory element binding factor 2	
Srgn	0	0	1	0	0	serglycin	
Srpk2	0	0	0	1	0	serine/arginine-rich protein specific kinase 2	
Srxn1	3	0	0	0	0	sulfiredoxin 1 homolog	
Ssbp2	0	0	0	0	1	single-stranded DNA binding protein 2	
Ssbp3	0	0	1	1	0	single-stranded DNA binding protein 3	
Sstr2	0	0	1	0	0	somatostatin receptor 2	
St6galnac4	0	0	2	0	0	GalNAc alpha-2,6-sialyltransferase	
St8sia2	0	0	1	0	0	ST8 alpha-N-acetyl-neuraminide alpha-2,8-sialyltransferase 2	
Stag1	0	0	0	1	1	stromal antigen 1	
Stat3	0	0	0	2	0	signal transducer and activator of transcription 3; APRF	89
Stch	0	0	0	1	0	stress 70 protein chaperone	
Stk25	0	0	0	1	0	serine/threonine kinase 25	
Stk3	0	0	0	1	0	serine/threonine kinase 3	
Stk40	1	0	0	0	0	serine/threonine kinase 40, SINK	
Stmn4	1	0	0	0	0	stathmin-like 4	
Stt3a	0	0	0	1	0	subunit of oligosaccharyltransferase complex, homolog A	
Stx1b2	0	0	0	1	0	syntaxin 1B	90
Stxbp5	0	0	0	0	1	syntaxin binding protein 5 (tomosyn)	
Sulf1	0	0	0	0	1	sulfatase 1	
Sult2b1	1	0	0	0	0	cytosolic sulfotransferase 2B1	
Sybl1	0	0	0	1	0	Vamp7	
Syncrip	0	0	0	3	0	synaptotagmin binding, cytoplasmic RNA interacting protein	
Synpo	1	0	0	0	1	synaptopodin	91
Syt12	0	0	1	0	0	synaptotagmin XII	
Syt4	0	0	0	1	0	synaptotagmin IV	92,93
Taf7	0	0	0	1	0	TAF7 RNA polymerase II	
Tagln2	0	0	0	0	1	transgelin 2	
Tagln3	0	0	1	0	0	transgelin 3	
Tanc1	1	0	1	0	0	tetratricopeptide repeat, ankyrin repeat and coiled-coil containing 1	
Tbc1d23	0	1	0	0	0	TBC1 domain family, member 23	
Tcea1	0	0	0	0	1	transcription elongation factor A (SII) 1	
Tceal8	1	0	0	0	0	transcription elongation factor A (SII)-like 8	
Tcp11l2	0	0	0	0	1	t-complex 11 (mouse) like 2	
Tfrc	0	0	0	5	0	transferrin receptor	

Tgfb2	0	1	0	0	0	transforming growth factor, beta 2	
Tgfbi	0	0	0	0	1	transforming growth factor, beta induced	
Tgfbr2	0	0	1	0	0	transforming growth factor, beta receptor 2	
Tgm1	0	0	0	0	1	transglutaminase 1, K polypeptide	
Tgm2	0	0	3	0	0	transglutaminase 2, C polypeptide	
Tgoln1	0	0	0	1	0	trans-golgi network protein; TGN38	
Thbs1	0	2	0	0	0	thrombospondin 1	
Thrap1	1	0	0	1	0	thyroid hormone receptor associated protein 1	
Thrap3	0	0	0	1	0	thyroid hormone receptor associated protein 3	
Timp1	0	0	0	0	1	tissue inhibitor of metalloproteinase 1	18
Tinf2	1	0	0	0	0	TERF1 (TRF1)-interact. nuclear factor 2	
Tiparp	3	0	0	0	0	TCDD-inducible poly(ADP-ribose) polymerase	
Tle3	0	2	0	0	0	transducin-like enhancer of split 3 (E(sp1) homolog	
Tll1	0	0	1	0	0	tld, tolloid, tolloid-like 1	
Tlr1	0	0	0	0	1	toll-like receptor 1	
Tlr2	0	0	0	0	1	toll-like receptor 2	
Tm4sf1	0	0	0	0	3	transmembrane 4 superfamily member 1	
Tm9sf3	0	0	0	1	0	transmembrane 9 superfamily member 3	
Tmbim1	0	0	0	1	0	transmembrane BAX inhibitor motif containing 1	
Tmed10	0	0	0	0	1	transmembrane emp24-like trafficking protein 10; Tmp21	
Tmed2	0	0	0	6	0	transmembrane emp24 domain trafficking protein 2	
Tmed7	0	0	0	1	1	transmembrane emp24 protein transport domain containing 7	
Tmeff1	0	0	1	0	0	transmembrane protein with EGF-like and two follistatin-like domains 1	
Tmem100	0	0	1	0	0	transmembrane protein 100	
Tmem178	1	0	0	0	0	transmembrane protein 178	
Tmem183a	0	0	0	1	0	transmembrane protein 183A	
Tmem2	3	0	0	0	0	transmembrane protein 2	
Tmem30a	0	0	0	0	1	transmembrane protein 30A	
Tmem33	0	0	0	1	0	transmembrane protein 33	
Tmem48	0	0	2	0	0	transmembrane protein 48; NDC1	
Tmem49	0	1	0	0	0	transmembrane protein 49; Vmp1	
Tmem90a	0	0	0	0	1	transmembrane protein 90A	
Tmepai	0	0	4	0	0	prostate transmembrane protein, androgen induced 1	
Tmod3	0	0	0	0	2	tropomodulin 3	
Tmpo	0	0	1	0	0	thymopoietin	
Tnc	0	0	0	1	0	tenascin C	94,95
Tnfrsf12a	0	0	2	0	0	tumor necrosis factor receptor superfamily, member 12a	
Tnfrsf1a	0	0	0	0	1	tumor necrosis factor receptor superfamily, member 1a	
Tnpo2	0	0	0	1	0	transportin 2 (importin 3)	
Tollip	0	0	0	1	0	toll interacting protein	
Tomm70a	0	0	0	1	0	translocase of outer mitochondrial membrane 70 homolog A	
Top2b	0	0	0	1	0	topoisomerase (DNA) II beta	
Tpbg	1	0	1	0	0	trophoblast glycoprotein	
Tpm1	1	0	0	0	0	tropomyosin 1, alpha	96
Tpm3	1	0	0	1	0	tropomyosin 3, gamma	
Trak1	0	1	0	0	0	trafficking protein, kinesin binding 1	
Trak2	0	0	0	0	2	trafficking protein, kinesin binding 2	
Trf	0	0	0	0	1	transferrin	
Trh	0	0	0	0	1	thyrotropin releasing hormone	22 ,97
Trib1	1	0	0	0	0	tribbles homolog 1	
Trib2	0	0	1	0	1	tribbles homolog 2	
Trim30	0	0	0	0	1	tripartite motif-containing 30	
Trim44	0	0	0	0	1	tripartite motif-containing 44	

Trim8	0	1	0	0	0	tripartite motif-containing 8	
Trim9	1	0	2	0	0	tripartite motif-containing 9;	
Trio	0	0	1	0	0	triple functional domain	
Trpc1	0	0	0	1	0	transient receptor potential cation channel, subfamily C, member 1	
Tsc22d1	0	1	0	0	0	TSC22 domain family, member 1	
Tsc22d2	1	0	1	0	0	TSC22 domain family, member 2	
Tsc22d3	0	2	0	0	0	TSC22 domain family, member 2	
Tspan18	1	0	0	0	0	tetraspanin 18	
Tspan4	0	0	0	0	1	tetraspanin 4; NAG-2	
Tspan5	0	0	0	1	0	tetraspanin 5	
Tspan9	0	0	1	0	0	tetraspanin 9	
Tspo	0	0	0	0	1	translocator protein	
Tubb2b	0	0	0	0	1	tubulin, beta 2b	
Tubb6	0	0	0	0	1	tubulin, beta 6	
Tulp4	1	1	1	0	0	tubby like protein 4	
Twf1	0	0	0	0	2	twinfilin, actin-binding protein, homolog 1	
Twsg1	0	0	0	1	0	twisted gastrulation homolog 1	
Txndc13	0	0	0	0	2	thioredoxin domain containing 13	
Ubc	1	0	0	0	0	ubiquitin C	
Ube2g1	0	0	0	1	0	ubiquitin-conjugating enzyme E2G 1	
Ube2q2	0	0	0	1	0	ubiquitin-conjugating enzyme E2Q 2	
Ube2v2	0	0	0	0	1	ubiquitin-conjugating enzyme E2 variant2	
Ube3a	0	0	0	1	0	ubiquitin protein ligase E3A	
Ubtd2	0	0	1	0	0	ubiquitin domain containing 2	
Ubtf	1	0	0	0	0	upstream binding transcription factor	
Ucp2	1	0	0	0	0	uncoupling protein 2; Slc25a8	98
Ugcg	0	2	0	0	0	UDP-glucose ceramide glucosyltransferase	
Ugdh	0	0	0	1	0	UDP-glucose dehydrogenase	
Upp1	0	0	0	0	1	uridine phosphorylase 1	
Usp18	0	0	0	0	1	ubiquitin specific peptidase 18	
Usp22	0	0	0	1	0	ubiquitin specific peptidase 22	
Usp24	0	0	0	1	0	ubiquitin specific peptidase 24	
Usp47	0	0	1	0	0	ubiquitin specific peptidase 47	
Usp53	0	1	0	0	0	ubiquitin specific peptidase 53	
Usp9x	0	0	0	3	0	deubiquitylating enzyme FAM/USP9X/DFFRX	
Utp18	0	0	0	0	1	small subunit processome component, homolog	
Uty	0	0	0	1	0	ubiquitously transcribed tetratricopeptide repeat gene, Y chromosome	
Vasp	0	0	0	1	0	vasodilator-stimulated phosphoprotein	
Vcl	0	2	0	0	0	vinculin	
Vegfa	0	0	1	0	0	vascular endothelial growth factor A	
Vgf	1	0	0	0	0	Vgf (VGF nerve growth factor inducible)	99-101
Vil2	0	0	0	1	0	villin 2, Ezr; ezrin	
Vim	0	0	0	0	3	vimentin	102
Vps35	0	0	0	0	1	vacuolar protein sorting 35 homolog	
Vps37a	0	0	1	0	0	vacuolar protein sorting 37A homolog	
Wdr1	0	0	0	3	0	WD repeat domain, actin interacting protein 1: AIP1	
Wdr57	0	0	0	0	1	WD repeat domain 57; Prp8bp	
Wisp1	0	0	1	0	0	WNT1 inducible signaling pathway protein 1; Elm1	
Wnt2	0	0	1	0	0	wingless-related MMTV integration site 2	
Wnt7b	0	0	0	0	2	wingless-related MMTV integration site 7B	
Wwtr1	0	0	3	0	0	WW domain containing transcription regulator 1	
Xbp1	0	0	3	0	0	X-box binding protein 1; TREB5	
Xdh	0	0	0	0	1	xanthine dehydrogenase	

Xpo1	0	0	0	0	1	exportin 1, CRM1 homolog	
Xpr1	0	0	0	2	0	xenotropic and polytropic retrovirus receptor 1; Rmc1	
Ythdf3	0	0	0	1	0	YTH domain family 3	
Ywhab	0	0	0	0	1	14-3-3 beta	
Ywhag	0	0	0	1	0	14-3-3gamma	
Ywhaz	0	2	0	2	0	14-3-3 zeta	103
Zc3h12c	2	1	0	0	0	zinc finger CCCH type containing 12C	
Zdhhc14	0	0	4	0	0	zinc finger, DHHC domain containing 14	
Zdhhc17	0	0	0	0	1	zinc finger, DHHC-type containing 17; huntingtin interacting protein 14	
Zdhhc18	0	0	1	0	0	zinc finger, DHHC domain containing 18	
Zdhhc2	0	0	1	1	0	zinc finger, DHHC domain containing 2	
Zfa	0	0	0	0	1	zinc finger protein, autosomal; Zfx	
Zfand5	0	1	0	2	0	zinc finger, AN1-type domain 5	
Zfml	0	0	1	0	0	zinc finger, matrin-like; Np220	
Zfp236	0	0	1	0	0	zinc finger protein 236	
Zfp260	0	0	0	0	1	zinc finger protein 260; Ozrf1, PEX1	
Zfp36	1	0	0	0	0	Tis11, zinc finger protein 36	
Zfp36l1	0	0	0	1	0	zinc finger protein 36, C3H type-like 1	
Zfp40	0	0	0	0	1	zinc finger protein 40; CTfin12	
Zfp451	0	0	2	0	0	zinc finger protein 451	
Zfp516	0	2	0	0	0	zinc finger protein 516	
Zfp521	0	0	0	0	1	zinc finger protein 521	
Zfp533	0	1	0	0	0	zinc finger protein 385B	
Zfp655	1	0	0	0	0	zinc finger protein 655	
Zfp703	0	0	1	0	0	zinc finger protein 703; Csmn1, End2	
Zfp709	0	0	0	0	1	zinc finger protein 709; KOX 6	
Zmiz1	1	0	2	0	0	zinc finger, MIZ-type containing 1; Rai17	
Zwint	2	2	0	0	0	ZW10 interactor	

Supplemtantal References (previous report of neuronal activity-induced expression in dataset S1)

1. Regard, J.B. *et al.*, Verge: a novel vascular early response gene. *J Neurosci* 24 (16), 4092-4103 (2004).
2. Panegyres, P.K. & Hughes, J., The neuroprotective effects of the recombinant interleukin-1 receptor antagonist rhIL-1ra after excitotoxic stimulation with kainic acid and its relationship to the amyloid precursor protein gene. *J Neurol Sci* 154 (2), 123-132 (1998).
3. Willoughby, D.A. *et al.*, Amyloid precursor protein mRNA encoding the Kunitz protease inhibitor domain is increased by kainic acid-induced seizures in rat hippocampus. *Exp Neurol* 118 (3), 332-339 (1992).
4. Link, W. *et al.*, Somatodendritic expression of an immediate early gene is regulated by synaptic activity. *Proc Natl Acad Sci U S A* 92 (12), 5734-5738 (1995).
5. Lyford, G.L. *et al.*, Arc, a growth factor and activity-regulated gene, encodes a novel cytoskeleton-associated protein that is enriched in neuronal dendrites. *Neuron* 14 (2), 433-445 (1995).
6. Nonaka, M. *et al.*, Kainic acid-induced seizure upregulates Na(+)/myo-inositol cotransporter mRNA in rat brain. *Brain Res Mol Brain Res* 70 (2), 179-186 (1999).
7. Zafra, F., Hengerer, B., Leibrock, J., Thoenen, H., & Lindholm, D., Activity dependent regulation of BDNF and NGF mRNAs in the rat hippocampus is mediated by non-NMDA glutamate receptors. *EMBO J* 9 (11), 3545-3550 (1990).
8. Dugich-Djordjevic, M.M. *et al.*, Regionally specific and rapid increases in brain-derived neurotrophic factor messenger RNA in the adult rat brain following seizures induced by systemic administration of kainic acid. *Neuroscience* 47 (2), 303-315 (1992).
9. Dugich-Djordjevic, M.M. *et al.*, BDNF mRNA expression in the developing rat brain following kainic acid-induced seizure activity. *Neuron* 8 (6), 1127-1138 (1992).

[10] Isackson, P.J., Huntsman, M.M., Murray, K.D., & Gall, C.M., BDNF mRNA expression is increased in adult rat forebrain after limbic seizures: temporal patterns of induction distinct from NGF. *Neuron* 6 (6), 937-948 (1991).
[11] Burazin, T.C. & Gundlach, A.L., Rapid and transient increases in cellular immediate early gene and neuropeptide mRNAs in cortical and limbic areas after amygdaloid kindling seizures in the rat. *Epilepsy Res* 26 (1), 281-293 (1996).
[12] Gubits, R.M., Burke, R.E., Casey-McIntosh, G., Bandele, A., & Munell, F., Immediate early gene induction after neonatal hypoxia-ischemia. *Brain Res Mol Brain Res* 18 (3), 228-238 (1993).
[13] Rozovsky, I. *et al.*, Selective expression of clusterin (SGP-2) and complement C1qB and C4 during responses to neurotoxins in vivo and in vitro. *Neuroscience* 62 (3), 741-758 (1994).
[14] Bu, J., Bruckner, S.R., Sengoku, T., Geddes, J.W., & Estus, S., Glutamate regulates caveolin expression in rat hippocampal neurons. *J Neurosci Res* 72 (2), 185-190 (2003).
[15] Manley, N.C., Bertrand, A.A., Kinney, K.S., Hing, T.C., & Sapolsky, R.M., Characterization of monocyte chemoattractant protein-1 expression following a kainate model of status epilepticus. *Brain Res* 1182, 138-143 (2007).
[16] Borges, K., McDermott, D.L., & Dingledine, R., Reciprocal changes of CD44 and GAP-43 expression in the dentate gyrus inner molecular layer after status epilepticus in mice. *Exp Neurol* 188 (1), 1-10 (2004).
[17] Mahata, S.K. *et al.*, Kainic acid seizures in the rat: differential expression of chromogranin A, carboxypeptidase H and peptidylglycine alpha-amidating monooxigenase in subfields of the hippocampal formation. *Acta Neuropathol* 86 (6), 590-595 (1993).
[18] Nedivi, E., Hevroni, D., Naot, D., Israeli, D., & Citri, Y., Numerous candidate plasticity-related genes revealed by differential cDNA cloning. *Nature* 363 (6431), 718-722 (1993).
[19] Theis, M., Si, K., & Kandel, E.R., Two previously undescribed members of the mouse CPEB family of genes and their inducible expression in the principal cell layers of the hippocampus. *Proc Natl Acad Sci U S A* 100 (16), 9602-9607 (2003).
[20] Konopka, D. *et al.*, Plasticity- and neurodegeneration-linked cyclic-AMP responsive element modulator/inducible cyclic-AMP early repressor messenger RNA expression in the rat brain. *Neuroscience* 86 (2), 499-510 (1998).
[21] Foradori, C.D., Lund, T.D., Nagahara, A.H., Koenig, J.I., & Handa, R.J., Corticotropin-releasing hormone heterogeneous nuclear RNA (hnRNA) and immunoreactivity are induced in extrahypothalamic brain sites by kainic-acid-induced seizures and are modulated by estrogen. *Brain Res* 1164, 44-54 (2007).
[22] Wilson, D.N. *et al.*, Microarray analysis of postictal transcriptional regulation of neuropeptides. *J Mol Neurosci* 25 (3), 285-298 (2005).
[23] Hertel, M., Tretter, Y., Alzheimer, C., & Werner, S., Connective tissue growth factor: a novel player in tissue reorganization after brain injury? *Eur J Neurosci* 12 (1), 376-380 (2000).
[24] Hevroni, D. *et al.*, Hippocampal plasticity involves extensive gene induction and multiple cellular mechanisms. *J Mol Neurosci* 10 (2), 75-98 (1998).
[25] Gass, P., Eckhardt, A., Schroder, H., Bravo, R., & Herdegen, T., Transient expression of the mitogen-activated protein kinase phosphatase MKP-1 (3CH134/ERP1) in the rat brain after limbic epilepsy. *Brain Res Mol Brain Res* 41 (1-2), 74-80 (1996).
[26] Kodama, M., Russell, D.S., & Duman, R.S., Electroconvulsive seizures increase the expression of MAP kinase phosphatases in limbic regions of rat brain. *Neuropsychopharmacology* 30 (2), 360-371 (2005).
[27] Li, L., Carter, J., Gao, X., Whitehead, J., & Tourtellotte, W.G., The neuroplasticity-associated arc gene is a direct transcriptional target of early growth response (Egr) transcription factors. *Mol Cell Biol* 25 (23), 10286-10300 (2005).
[28] Honkaniemi, J. & Sharp, F.R., Prolonged expression of zinc finger immediate-early gene mRNAs and decreased protein synthesis following kainic acid induced seizures. *Eur J Neurosci* 11 (1), 10-17 (1999).

[29] Flood, W.D., Moyer, R.W., Tsykin, A., Sutherland, G.R., & Koblar, S.A., Nxf and Fbxo33: novel seizure-responsive genes in mice. *Eur J Neurosci* 20 (7), 1819-1826 (2004).

[30] Van Der Wal, E.A., Gomez-Pinilla, F., & Cotman, C.W., Seizure-associated induction of basic fibroblast growth factor and its receptor in the rat brain. *Neuroscience* 60 (2), 311-323 (1994).

[31] Beer, J., Mielke, K., Zipp, M., Zimmermann, M., & Herdegen, T., Expression of c-jun, junB, c-fos, fra-1 and fra-2 mRNA in the rat brain following seizure activity and axotomy. *Brain Res* 794 (2), 255-266 (1998).

[32] Morgan, J.I., Cohen, D.R., Hempstead, J.L., & Curran, T., Mapping patterns of c-fos expression in the central nervous system after seizure. *Science* 237 (4811), 192-197 (1987).

[33] Bing, G. *et al.*, Long-term expression of Fos-related antigen and transient expression of delta FosB associated with seizures in the rat hippocampus and striatum. *J Neurochem* 68 (1), 272-279 (1997).

[34] Brackmann, M., Zhao, C., Kuhl, D., Manahan-Vaughan, D., & Braunewell, K.H., MGluRs regulate the expression of neuronal calcium sensor proteins NCS-1 and VILIP-1 and the immediate early gene arg3.1/arc in the hippocampus in vivo. *Biochem Biophys Res Commun* 322 (3), 1073-1079 (2004).

[35] Genin, A. *et al.*, Regulated expression of the neuronal calcium sensor-1 gene during long-term potentiation in the dentate gyrus in vivo. *Neuroscience* 106 (3), 571-577 (2001).

[36] Meyer, A. *et al.*, Kainic acid increases the expression of the prohormone convertases furin and PC1 in the mouse hippocampus. *Brain Res* 732 (1-2), 121-132 (1996).

[37] Sohl, G. *et al.*, Expression of connexin genes in hippocampus of kainate-treated and kindled rats under conditions of experimental epilepsy. *Brain Res Mol Brain Res* 83 (1-2), 44-51 (2000).

[38] Reeben, M., Laurikainen, A., Hiltunen, J.O., Castren, E., & Saarma, M., The messenger RNAs for both glial cell line-derived neurotrophic factor receptors, c-ret and GDNFRalpha, are induced in the rat brain in response to kainate-induced excitation. *Neuroscience* 83 (1), 151-159 (1998).

[39] Condorelli, D.F., Trovato-Salinaro, A., Mudo, G., Mirone, M.B., & Belluardo, N., Cellular expression of connexins in the rat brain: neuronal localization, effects of kainate-induced seizures and expression in apoptotic neuronal cells. *Eur J Neurosci* 18 (7), 1807-1827 (2003).

[40] Mathern, G.W., Pretorius, J.K., Mendoza, D., Lozada, A., & Kornblum, H.I., Hippocampal AMPA and NMDA mRNA levels correlate with aberrant fascia dentata mossy fiber sprouting in the pilocarpine model of spontaneous limbic epilepsy. *J Neurosci Res* 54 (6), 734-753 (1998).

[41] Bottai, D. *et al.*, Synaptic activity-induced conversion of intronic to exonic sequence in Homer 1 immediate early gene expression. *J Neurosci* 22 (1), 167-175 (2002).

[42] Brakeman, P.R. *et al.*, Homer: a protein that selectively binds metabotropic glutamate receptors. *Nature* 386 (6622), 284-288 (1997).

[43] Kato, A., Ozawa, F., Saitoh, Y., Hirai, K., & Inokuchi, K., vesl, a gene encoding VASP/Ena family related protein, is upregulated during seizure, long-term potentiation and synaptogenesis. *FEBS Lett* 412 (1), 183-189 (1997).

[44] Elliott, R.C., Khademi, S., Pleasure, S.J., Parent, J.M., & Lowenstein, D.H., Differential regulation of basic helix-loop-helix mRNAs in the dentate gyrus following status epilepticus. *Neuroscience* 106 (1), 79-88 (2001).

[45] Newton, S.S., Girgenti, M.J., Collier, E.F., & Duman, R.S., Electroconvulsive seizure increases adult hippocampal angiogenesis in rats. *Eur J Neurosci* 24 (3), 819-828 (2006).

[46] Pennypacker, K.R. *et al.*, Kainate-induced changes in opioid peptide genes and AP-1 protein expression in the rat hippocampus. *J Neurochem* 60 (1), 204-211 (1993).

[47] Minami, M. *et al.*, Kainic acid induces leukemia inhibitory factor mRNA expression in the rat brain: differences in the time course of mRNA expression between the dentate gyrus and hippocampal CA1/CA3 subfields. *Brain Res Mol Brain Res* 107 (1), 39-46 (2002).

[48] Trifunovski, A. *et al.*, Neuronal activity-induced regulation of Lingo-1. *Neuroreport* 15 (15), 2397-2400 (2004).
[49] Hinks, G.L. *et al.*, Expression of LIM protein genes Lmo1, Lmo2, and Lmo3 in adult mouse hippocampus and other forebrain regions: differential regulation by seizure activity. *J Neurosci* 17 (14), 5549-5559 (1997).
[50] Shinoda, S. *et al.*, Formation of a tumour necrosis factor receptor 1 molecular scaffolding complex and activation of apoptosis signal-regulating kinase 1 during seizure-induced neuronal death. *Eur J Neurosci* 17 (10), 2065-2076 (2003).
[51] Vician, L.J. *et al.*, MAPKAP kinase-2 is a primary response gene induced by depolarization in PC12 cells and in brain. *J Neurosci Res* 78 (3), 315-328 (2004).
[52] Emerson, M.R., Samson, F.E., & Pazdernik, T.L., Effects of hypoxia preconditioning on expression of metallothionein-1,2 and heme oxygenase-1 before and after kainic acid-induced seizures. *Cell Mol Biol (Noisy-le-grand)* 46 (3), 619-626 (2000).
[53] Pei, Q., Burnet, P.J., & Zetterstrom, T.S., Changes in mRNA abundance of microtubule-associated proteins in the rat brain following electroconvulsive shock. *Neuroreport* 9 (3), 391-394 (1998).
[54] Pollard, H., Khrestchatisky, M., Moreau, J., Ben-Ari, Y., & Represa, A., Correlation between reactive sprouting and microtubule protein expression in epileptic hippocampus. *Neuroscience* 61 (4), 773-787 (1994).
[55] Ohkawa, N. *et al.*, Molecular cloning and characterization of neural activity-related RING finger protein (NARF): a new member of the RBCC family is a candidate for the partner of myosin V. *J Neurochem* 78 (1), 75-87 (2001).
[56] Matsui, F. *et al.*, Transient expression of juvenile-type neurocan by reactive astrocytes in adult rat brains injured by kainate-induced seizures as well as surgical incision. *Neuroscience* 112 (4), 773-781 (2002).
[57] Lin, Y. *et al.*, Activity-dependent regulation of inhibitory synapse development by Npas4. *Nature* 455 (7217), 1198-1204 (2008).
[58] O'Brien, R.J. *et al.*, Synaptic clustering of AMPA receptors by the extracellular immediate-early gene product Narp. *Neuron* 23 (2), 309-323 (1999).
[59] Reti, I.M. & Baraban, J.M., Sustained increase in Narp protein expression following repeated electroconvulsive seizure. *Neuropsychopharmacology* 23 (4), 439-443 (2000).
[60] Silva, A.P. *et al.*, Up-regulation of neuropeptide Y levels and modulation of glutamate release through neuropeptide Y receptors in the hippocampus of kainate-induced epileptic rats. *J Neurochem* 93 (1), 163-170 (2005).
[61] French, P.J. *et al.*, Seizure-induced gene expression in area CA1 of the mouse hippocampus. *European Journal of Neuroscience* 14 (12), 2037-2041 (2001).
[62] Maruyama, K. *et al.*, The NGFI-B subfamily of the nuclear receptor superfamily (review). *Int J Oncol* 12 (6), 1237-1243 (1998).
[63] Ponnio, T. & Conneely, O.M., nor-1 regulates hippocampal axon guidance, pyramidal cell survival, and seizure susceptibility. *Mol Cell Biol* 24 (20), 9070-9078 (2004).
[64] Nedivi, E., Wu, G.Y., & Cline, H.T., Promotion of dendritic growth by CPG15, an activity-induced signaling molecule. *Science* 281 (5384), 1863-1866 (1998).
[65] Dugich-Djordjevic, M.M. *et al.*, Differential regulation of catalytic and non-catalytic trkB messenger RNAs in the rat hippocampus following seizures induced by systemic administration of kainate. *Neuroscience* 66 (4), 861-877 (1995).
[66] Yamagata, K. *et al.*, Arcadlin is a neural activity-regulated cadherin involved in long term potentiation. *J Biol Chem* 274 (27), 19473-11979 (1999).
[67] Kim, H.C. *et al.*, Dextromethorphan blocks opioid peptide gene expression in the rat hippocampus induced by kainic acid. *Neuropeptides* 31 (2), 105-112 (1997).
[68] Lason, W., Przewlocka, B., & Przewlocki, R., The effects of excitatory amino acids on proenkephalin and prodynorphin mRNA levels in the hippocampal dentate gyrus of the rat; an in situ hybridization study. *Brain Res Mol Brain Res* 12 (1-3), 243-247 (1992).

69 Feldman, J.D. *et al.*, Seizure activity induces PIM-1 expression in brain. *J Neurosci Res* 53 (4), 502-509 (1998).
70 Konietzko, U. *et al.*, Pim kinase expression is induced by LTP stimulation and required for the consolidation of enduring LTP. *EMBO J* 18 (12), 3359-3369 (1999).
71 Valente, T. *et al.*, Zac1 is up-regulated in neural cells of the limbic system of mouse brain following seizures that provoke strong cell activation. *Neuroscience* 128 (2), 323-336 (2004).
72 Qian, Z., Gilbert, M.E., Colicos, M.A., Kandel, E.R., & Kuhl, D., Tissue-Plasminogen Activator Is Induced as an Immediate Early Gene during Seizure, Kindling and Long-Term Potentiation. *Nature* 361 (6411), 453-457 (1993).
73 Yoshida, S. & Shiosaka, S., Plasticity-related serine proteases in the brain (review). *Int J Mol Med* 3 (4), 405-409 (1999).
74 Kauselmann, G. *et al.*, The polo-like protein kinases Fnk and Snk associate with a Ca(2+)- and integrin-binding protein and are regulated dynamically with synaptic plasticity. *EMBO J* 18 (20), 5528-5539 (1999).
75 Lee, B., Dziema, H., Lee, K.H., Choi, Y.S., & Obrietan, K., CRE-mediated transcription and COX-2 expression in the pilocarpine model of status epilepticus. *Neurobiol Dis* 25 (1), 80-91 (2007).
76 Ravizza, T. *et al.*, Dynamic induction of the long pentraxin PTX3 in the CNS after limbic seizures: evidence for a protective role in seizure-induced neurodegeneration. *Neuroscience* 105 (1), 43-53 (2001).
77 Palm, K., Belluardo, N., Metsis, M., & Timmusk, T., Neuronal expression of zinc finger transcription factor REST/NRSF/XBR gene. *J Neurosci* 18 (4), 1280-1296 (1998).
78 Ingi, T. *et al.*, Dynamic regulation of RGS2 suggests a novel mechanism in G-protein signaling and neuronal plasticity. *J Neurosci* 18 (18), 7178-7188 (1998).
79 Gold, S.J., Heifets, B.D., Pudiak, C.M., Potts, B.W., & Nestler, E.J., Regulation of regulators of G protein signaling mRNA expression in rat brain by acute and chronic electroconvulsive seizures. *J Neurochem* 82 (4), 828-838 (2002).
80 Gold, S.J., Ni, Y.G., Dohlman, H.G., & Nestler, E.J., Regulators of G-protein signaling (RGS) proteins: region-specific expression of nine subtypes in rat brain. *J Neurosci* 17 (20), 8024-8037 (1997).
81 Yamagata, K. *et al.*, Rheb, a Growth Factor-Regulated and Synaptic Activity-Regulated Gene, Encodes a Novel Ras-Related Protein. *Journal of Biological Chemistry* 269 (23), 16333-16339 (1994).
82 Marti, E., Blasi, J., & Ferrer, I., Early induction of secretoneurin expression following kainic acid administration at convulsant doses in the rat and gerbil hippocampus. *Hippocampus* 12 (2), 174-185 (2002).
83 Masos, T. & Miskin, R., mRNAs encoding urokinase-type plasminogen activator and plasminogen activator inhibitor-1 are elevated in the mouse brain following kainate-mediated excitation. *Brain Res Mol Brain Res* 47 (1-2), 157-169 (1997).
84 Shimizu-Nishikawa, K., Kajiwara, K., Kimura, M., Katsuki, M., & Sugaya, E., Cloning and expression of SEZ-6, a brain-specific and seizure-related cDNA. *Brain Res Mol Brain Res* 28 (2), 201-210 (1995).
85 Rosell, D.R., Akama, K.T., Nacher, J., & McEwen, B.S., Differential expression of suppressors of cytokine signaling-1, -2, and -3 in the rat hippocampus after seizure: implications for neuromodulation by gp130 cytokines. *Neuroscience* 122 (2), 349-358 (2003).
86 Hermey, G. *et al.*, The three sorCS genes are differentially expressed and regulated by synaptic activity. *J Neurochem* 88 (6), 1470-1476 (2004).
87 Sun, W. *et al.*, Identification of novel electroconvulsive shock-induced and activity-dependent genes in the rat brain. *Biochem Biophys Res Commun* 327 (3), 848-856 (2005).
88 Kim, S.Y. *et al.*, Osteopontin in kainic acid-induced microglial reactions in the rat brain. *Mol Cells* 13 (3), 429-435 (2002).

[89] Choi, J.S. *et al.*, Upregulation of gp130 and differential activation of STAT and p42/44 MAPK in the rat hippocampus following kainic acid-induced seizures. *Brain Res Mol Brain Res* 119 (1), 10-18 (2003).
[90] Kamphuis, W. *et al.*, The expression of syntaxin1B/GR33 mRNA is enhanced in the hippocampal kindling model of epileptogenesis. *J Neurochem* 65 (5), 1974-1980 (1995).
[91] Roth, S.U., Sommer, C., Mundel, P., & Kiessling, M., Expression of synaptopodin, an actin-associated protein, in the rat hippocampus after limbic epilepsy. *Brain Pathol* 11 (2), 169-181 (2001).
[92] Tocco, G. *et al.*, Two synaptotagmin genes, Syt1 and Syt4, are differentially regulated in adult brain and during postnatal development following kainic acid-induced seizures. *Brain Res Mol Brain Res* 40 (2), 229-239 (1996).
[93] Vician, L. *et al.*, Synaptotagmin IV is an immediate early gene induced by depolarization in PC12 cells and in brain. *Proc Natl Acad Sci U S A* 92 (6), 2164-2168 (1995).
[94] Ferhat, L., Chevassus-Au-Louis, N., Khrestchatisky, M., Ben-Ari, Y., & Represa, A., Seizures induce tenascin-C mRNA expression in neurons. *J Neurocytol* 25 (9), 535-546 (1996).
[95] Nakic, M., Mitrovic, N., Sperk, G., & Schachner, M., Kainic acid activates transient expression of tenascin-C in the adult rat hippocampus. *J Neurosci Res* 44 (4), 355-362 (1996).
[96] Sussman, M.A. *et al.*, Neural tropomodulin: developmental expression and effect of seizure activity. *Brain Res Dev Brain Res* 80 (1-2), 45-53 (1994).
[97] Kreider, M.S., Wolfinger, B.L., & Winokur, A., Systemic administration of kainic acid produces elevations in TRH in rat central nervous system. *Regul Pept* 28 (1), 83-93 (1990).
[98] Clavel, S., Paradis, E., Ricquier, D., & Richard, D., Kainic acid upregulates uncoupling protein-2 mRNA expression in the mouse brain. *Neuroreport* 14 (16), 2015-2017 (2003).
[99] Possenti, R., Di Rocco, G., Nasi, S., & Levi, A., Regulatory elements in the promoter region of vgf, a nerve growth factor-inducible gene. *Proc Natl Acad Sci U S A* 89 (9), 3815-3819 (1992).
[100] Salton, S.R., Fischberg, D.J., & Dong, K.W., Structure of the gene encoding VGF, a nervous system-specific mRNA that is rapidly and selectively induced by nerve growth factor in PC12 cells. *Mol Cell Biol* 11 (5), 2335-2349 (1991).
[101] Snyder, S.E. *et al.*, The messenger RNA encoding VGF, a neuronal peptide precursor, is rapidly regulated in the rat central nervous system by neuronal activity, seizure and lesion. *Neuroscience* 82 (1), 7-19 (1998).
[102] Jorgensen, M.B. *et al.*, Microglial and astroglial reactions to ischemic and kainic acid-induced lesions of the adult rat hippocampus. *Exp Neurol* 120 (1), 70-88 (1993).
[103] Schindler, C.K., Heverin, M., & Henshall, D.C., Isoform- and subcellular fraction-specific differences in hippocampal 14-3-3 levels following experimentally evoked seizures and in human temporal lobe epilepsy. *J Neurochem* 99 (2), 561-569 (2006).
